# Transcriptome analysis reveals a stress response of *Shewanella oneidensis* deprived of background levels of ionizing radiation

**DOI:** 10.1371/journal.pone.0196472

**Published:** 2018-05-16

**Authors:** Hugo Castillo, Xiaoping Li, Faye Schilkey, Geoffrey B. Smith

**Affiliations:** 1 Department of Biology, New Mexico State University, Las Cruces, NM, United States of America; 2 Department of Botany and Plant Pathology, Oregon State University, Hermiston, OR, United States of America; 3 National Center for Genome Resources, Santa Fe, NM, United States of America; Chuo University, JAPAN

## Abstract

Natural ionizing background radiation has exerted a constant pressure on organisms since the first forms of life appeared on Earth, so that cells have developed molecular mechanisms to avoid or repair damages caused directly by radiation or indirectly by radiation-induced reactive oxygen species (ROS). In the present study, we investigated the transcriptional effect of depriving *Shewanella oneidensis* cultures of background levels of radiation by growing the cells in a mine 655 m underground, thus reducing the dose rate from 72.1 to 0.9 nGy h^-1^ from control to treatment, respectively. RNASeq transcriptome analysis showed the differential expression of 4.6 and 7.6% of the *S*. *oneidensis* genome during early- and late-exponential phases of growth, respectively. The greatest change observed in the treatment was the downregulation of ribosomal proteins (21% of all annotated ribosomal protein genes during early- and 14% during late-exponential) and tRNA genes (14% of all annotated tRNA genes in early-exponential), indicating a marked decrease in protein translation. Other significant changes were the upregulation of membrane transporters, implying an increase in the traffic of substrates across the cell membrane, as well as the up and downregulation of genes related to respiration, which could be interpreted as a response to insufficient oxidants in the cells. In other reports, there is evidence in multiple species that some ROS not just lead to oxidative stress, but act as signaling molecules to control cellular metabolism at the transcriptional level. Consistent with these reports, several genes involved in the metabolism of carbon and biosynthesis of amino acids were also regulated, lending support to the idea of a wide metabolic response. Our results indicate that *S*. *oneidensis* is sensitive to the withdrawal of background levels of ionizing radiation and suggest that a transcriptional response is required to maintain homeostasis and retain normal growth.

## Introduction

Natural ionizing radiation is a constant, pervasive environmental factor that plays a role in the biology of all organisms on Earth. Commonly referred to as “background radiation”, it is a collective term to describe cosmic, terrestrial, and internal sources of different forms of energy and electrically charged particles [1]. Also, background radiation varies widely geographically mainly due to the radioisotopes content of soil, rocks, and altitude [2]. As a result, all forms of radiation have different abilities to interact with matter, transferring energy and potentially causing the disruption of chemical bonds in a variety of molecules. In cells, the release of energy by an alpha or beta particle, or by a gamma ray results in the conformational change of biomolecules such as nucleic acids, lipids, and proteins, either by direct ionization events or through the formation of free radicals, also known as reactive oxygen species (ROS) [2, 3]. However, ever since their appearance on Earth approximately 3.5 billion years ago [4], cells have developed sensing and defense mechanisms in response to the insult of radiation, such as the production of antioxidants, the expression of ROS (Reactive oxygen species)-scavenging enzymes, and DNA repair systems [5]. In the light of these adaptive responses, it is fair to question if after millions of years of selective pressure by ionizing radiation, some of its products (ROS) have come to play an essential role in the biology of organisms. If so, the absence of background levels of radiation would elicit a specific stress response. In support of this hypothesis, there is growing evidence that some ROS, such as hydrogen peroxide and superoxide ions, act as regulatory components of biological processes such as growth, ROS homeostasis, antioxidant gene regulation, and DNA repair, among others [6–9]. Exposing cells to below-background doses of radiation might result in what can be construed as a stress response in different species, such as the inhibition of normal growth rate in *Paramecium tetraurelia* and *Synechococcus lividus* [10]; the decreased protection to radiomimetic agents in *Saccharomyces cerevisiae* [11]; a higher sensitivity to gamma rays and apoptosis in *Cricetulus griseus* V79 cells [12]; the lower cell density in *Mus musculus* L5178Y cells [13]; the regulation in the activity of stress-related enzymes in *Cricetulus griseus* V79 cells [14]; and the regulation of DNA repair and oxidative stress genes in *Shewanella oneidensis* and *Deinococcus radiodurans* [15–17]. Thus, the growing body of knowledge suggests that regardless of differences in cellular complexity and physiology, organisms exert a stress response to this peculiar radiation deprivation treatment. We report here the first transcriptome of an organism responding to the absence of natural levels of radiation and document this response in the bacterium *S*. *oneidensis*. Although our treatment simulates an artificial condition non-existent in any biologically-relevant place on Earth, it provides valuable data that suggest a role for ionizing radiation on the development of present-day bacteria.

*Shewanella oneidensis* is a facultative anaerobe, a Gram-negative bacterium, estimated to be 10 and 143 times more sensitive to ionizing radiation than *E*. *coli* and *D*. *radiodurans*, respectively [18]. Transcriptome analysis upon acute exposure to ionizing radiation [19], UVABC rays [20], and solar radiation [21] show the induction of systems aimed to combat oxidative stress, to protect the cells from protein damage, and to secrete radiation damage byproducts through multidrug and heavy metal efflux pumps, among others. Our transcriptome analysis of the removal of background levels of radiation from *S*. *oneidensis* shows a growth phase-dependent gene regulation response to this unusual environmental cue. During early exponential growth, a significant number of ribosomal proteins and tRNA-coding genes are downregulated in the below-background dosage group; while the late-exponential phase is characterized mainly by the upregulation of genes related to membrane transport, oxidative phosphorylation, and biosynthesis of amino acids, as well as the downregulation of genes involved in protein folding. These patterns of regulation indicate that *S*. *oneidensis* reacts to the absence of background levels of ionizing radiation in a way that resembles a response to different types of environmental stress, suggesting that cells have not only adapted to thrive in the presence of environmental radiation but have also developed a certain degree of “dependence” on it to maintain homeostasis.

## Materials and methods

### LBRE (Low Background Radiation Experiment) laboratory, radiation treatments and dosimetry

**Fig 1:**
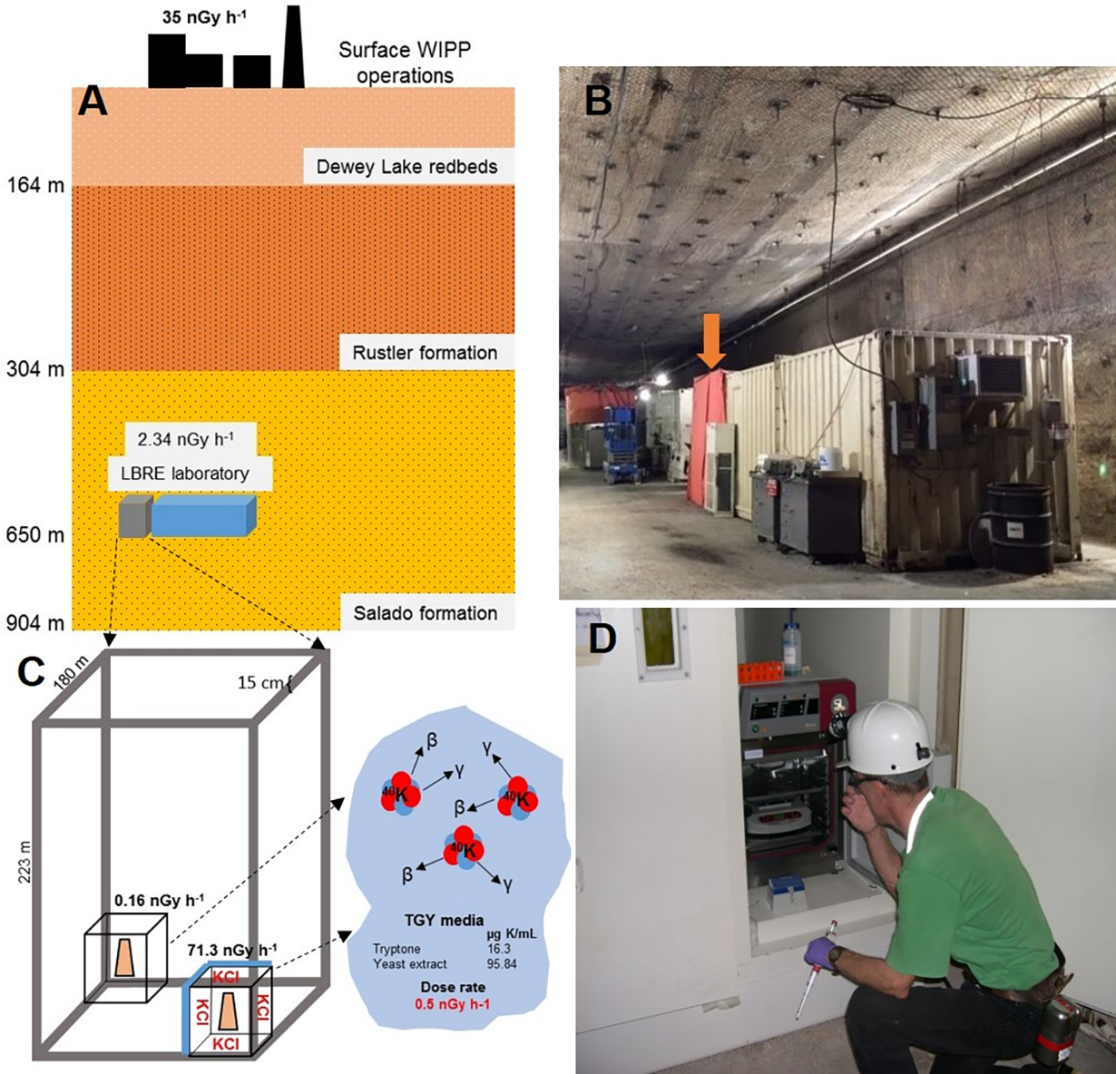
Shielding from background radiation at the LBRE laboratory at the Waste Isolation Pilot Plant. (A) The LBRE laboratory, (hosted by the Waste Isolation Pilot Plant (WIPP), a nuclear waste repository near Carlsbad, NM.), is located in the middle of the 600 m-thick Salado formation. (B) Laboratories underground at WIPP are conex shipping containers modified to accommodate office and laboratory equipment. The LBRE steel vault used in this study is under the orange fire blanket (arrow) to provide insulation from the high temperature in the drift. Inside the vault there are two incubators, one of which has 11.5 kg of KCl as a source of ~185 kBq of potassium-40 to ‘add-back” natural levels of radiation and act as our experimental control. Potassium is also present in the media in both incubators adding a dose rate of about 0.5 nGy mL^-1^ h^-1^ to both the treatment and control. (D) Sampling (Geoffrey Smith) from the plus KCl control incubator (70 nGy h^-1^) inside the vault made from pre-World War II steel.

The LBRE (Low Background Radiation Experiment) laboratory is located at a depth of 655 m at the Waste Isolation Pilot Plant (WIPP) near Carlsbad, NM, within the Salado formation (Fig 1A). Protection from cosmic rays and related particles by the rock overburden and the very low abundance of uranium-238, thorium-232, and pottasium-40 radioisotopes in halite, the most abundant mineral at this depth [22], naturally lowered background radiation by a factor of four [16]. Additionally, the incubators used for the LBRE experiments were further shielded by a 15 cm thick low-activity steel vault built from fallout-free pre-World War II materials. For the control incubator, we aimed for a gamma dose rate of 100 nGy h^-1^ based on the U.S. Nuclear Regulatory Commission (NRC) estimate of natural background exposure being 347 nGy h^-1^ [23], and a UK study reporting the proportion of natural background radiation that came from gamma exposure as 94.7 nGy h^-1^ [24]. In order to simulate this dose rate, the inside surfaces of the control incubator were lined with 11.5 kg of KCl (equivalent to 185 kBq of potassium-40) uniformly distributed in plastic containers (Fig 1A and 1C). Radiation dosimetry using a NaI detector and Monte-Carlo N-Particle (MCNP) analysis estimated the dose rate to be 0.16 and 71.3 nGy h^-1^ in the treatment and control incubators, respectively (for more details, see [16]). Radon concentration inside the vault was measured with a SafetySiren™ Pro Series 3 detector at 15.6 ± 4 Bq m^3^, in agreement with measurements made by the EXO (http://www-project.slac.stanford.edu/exo), a particle physics experiment also hosted at WIPP (Andrea Pocar, personal communication). Using a range of values from 7 to 31 Bq m^3^ as input for MCNP estimates in our 5.2 × 10^4^ cm^3^ incubators, radon concentration ranged from 1 to 4 pGy h^-1^. This contribution was included in our estimated total dose rate of 0.16 nGy h^-1^ (excluding sources in the growth media) in the treatment incubator, as previously reported [17]. Considering the potassium-40 in the 1.5 mL of growth medium used (0.5 nGy h^-1^ per mL) this gives a dose rate of 0.91 and 72.05 nGy h^-1^ in the treatment and control, respectively. For more details, see [16].

### Bacterial growth conditions

For each of three biological replicates, seed cultures of *S*. *oneidensis* MR-1 (ATCC 700550) were grown at the WIPP surface laboratory from a single colony in 2 mL of pH 7.0 TGY broth (5 g tryptone, 3 g yeast extract, and 1 g glucose, per L) under constant agitation (150 rpm) during 72 hours. An aliquot of 20 μL of this culture was used to inoculate 2 mL of broth to start an overnight culture under the same culturing conditions. The overnight culture was then transported at room temperature to the underground laboratory and diluted to approximately 3×10^7^ cells per mL to initiate the experiment. Initially, 1.5 mL of cell suspension were transferred into the top row of the 24-well plate and grown in the below-background (treatment) and background (control) incubators at 200 rpm during 24 hours at 30°C. After this initial growth period, a sample pooled from the first 4 wells was then diluted 1:50 and 1.5 mL aliquots were transferred to the second row of the plate to re-initiate their growth under their respective conditions.

### Growth measurement and sampling

The growth dynamics of the cultures was followed by measuring optical density at 630 nm on an ELX800 microplate reader (Biotek, Winooski, VT, USA) at 5, 8, 13, 17, and 24 hours. In parallel, 300 μL of culture from duplicate wells were transferred into 600 μL of RNAprotect solution (QIAGEN, Valencia, CA, USA) for RNA stabilization and storage, processed according to the manufacturer’s instructions, and stored at -20°C.

### RNA extraction, libraries preparation and sequencing

Total RNA was extracted from RNA-protected samples using the RNAeasy QIAGEN kit (QIAGEN, Valencia, CA, USA) following the enzymatic lysis and proteinase K digestion protocol, as instructed by the manufacturer’s instructions and including an additional treatment with DNAse I (QIAGEN, Valencia, CA, USA) to remove traces of genomic DNA. Total RNA concentration was measured using the RNA Qubit assay (Invitrogen, Burlington, ON, Canada) and RNA integrity was evaluated using the Bioanalyzer RNA pico Assay (Agilent Technologies, Santa Clara, CA, USA) following the manufacturer’s protocol. Depletion of the rRNA was performed using the RiboZero kit (Bacteria) and the rRNA-depleted samples were purified using the RNAClean XP kit (Beckam Coulter, Beverly, MA, USA). Libraries were constructed using the ScriptSeq Complete kit for bacteria from (Epicentre, Madison, WI, USA). In brief, rRNA-depleted RNA fragmentation and the addition of the cDNA synthesis primer were performed by incubation in the fragmentation buffer at 85°C for 5 minutes, followed by the synthesis of the cDNA and the 3’-terminal tagging of the cDNA. Prior to PCR amplification, the cDNA was purified using the Agencourt AMPure XP system (Beckman Coulter, Beverly, MA, USA). The second strand cDNA was generated by adding the Illumina adapters as the forward primer and a ScriptSeq index primer as the reverse primer to allow multiplexing during sequencing. The resulting RNASeq libraries were purified using the AMPure XP system (Beckman Coulter, Beverly, MA, USA), quantified with the DNA Qubit assay, and their quality evaluated using the Bioanalyzer High Sensitivity DNA assay (Agilent Technologies, Santa Clara, CA, USA) to confirm the adequate distribution of fragments length. Fifty bp single reads were generated using the Illumina HiSeq 2000 platform at the National Center for Genome Research in Santa Fe, NM.

### Bioinformatic analysis

The reads were mapped against the *S*. *oneidensis* reference genome (NC_004347 and NC_004349) using EDGE-pro v1.3.1 (Estimated degree of gene expression in prokaryotic genomes) software [25]. In brief, the FASTA (*.fna), protein table (*.ptt), and rRNA/tRNA genes coordinates (*.rnt) files corresponding to the chromosome (NC_004347) and megaplasmid (NC_004349) units of *S*. *oneidensis* were concatenated prior to the alignment. The alignment on EDGE-pro was run using the default parameters. The differential gene expression analysis was performed with edgeR (version 1) using as input the count table generated by EDGE-pro. Only genes with a fold change ≥ 2 and an FDR-value ≤ 0.10 were considered significant for the purpose of this study. The reads from both 5 and 13 h libraries were deposited in the NCBI SRA depository under the accession number PRJNA396034. The significantly up- and down-regulated genes were analyzed for gene ontology (GO) term enrichment (*p* value ≤ 0.05), separately, using GOToolBox (http://genome.crg.es/GOToolBox/), and the significantly enriched terms further explored on the REVIGO web application [26] to identify and visualize relationships among the GO terms.

### RT-qPCR

The validity of the differential expression was tested using RT-qPCR for direct comparison with RNASeq. Prior to RT-PCR, cDNA was synthesized with the iScript™ Reverse Transcription Supermix for RT-qPCR (BioRad, Hercules, CA, USA) using total RNA as template and a reaction incubation program of priming at 25°C for 5 min, reverse transcription at 46°C for 20 min, and RT inactivation at 95°C for 1 min. The qPCR reactions (10 uL) were performed in triplicate using the SsoAdvanced Universal SYBR Green Supermix (BioRad, Hercules, CA, USA), 0.5 μM of each primer (Table 1), and 1 ng of cDNA as template. The PCR program was as follows: Polymerase activation and DNA denaturation step of 30 s at 98°C, followed by 40 cycles of denaturation at 98°C for 15 s and primer annealing/extension at 60°C for 30 s. After amplification, a melting curve analysis of 60 cycles from 65 to 95°C at a rate of 0.5°C per cycle was included to assess the specificity of the amplification. The relative expression of the target genes *was* calculated using *gyrA and gyrB* as reference genes and using the efficiency-corrected model [27]. For each comparison, 9 to 12 C_t_ values from three biological replicates were used for all calculations.

**Table 1:** Primers used for the validation of the transcriptome analysis on early and late-exponential *S*. *oneidensis* cultures.

Gene	Function	Sequence (5'-3')		
		Forward	Reverse	Size (bp)
*SO0760*	Ammonium transporter	CCTGTTGAAGGTTACTGGAC	CCCAGTAATAATACCCCAGC	128
*SO0074*	ABC-type efflux system permease component	AGTGTCGGTGTGTTGCTCTG	GCCCTAATACCAAGGCACAA	117
*rpsQ*	Small subunit ribosomal protein S17	CGTACGACTAAGATCCATGC	CCAGGGTCCAAGATTTAGTC	109
*omp35*	Outer membrane porin	ATTAGCACTGGCCTCATTCG	CGTTAGTGCCAGATTGCAGA	113
*liuR*	Transcriptional repressor	CAACTAACACGCCACAAACG	ACTTGAGTCGCACCCTGTCT	162
*groES*	10 kDa chaperonin	CGCGTAATCGTTAAGCGTCT	CCCACTTTCACATCCAGAGG	161
*rplK*	50S ribosomal protein L11	TCTCCACGTCCAAACACTCA	ATTGAACGCGCAGTACCTTC	140
*groEL*	60 kDa chaperonin	TGACGTTGAAGTGGCTAACG	GCCACAACAGAGGCTTCTTC	117
*SO3545*	Outer membrane porin	ATCTAGGTAGTGCTGACTGG	GATAGGCTCCAACGGTTAC	112
*rplT*	50S ribosomal protein L20	GGTTATTATGGCGCTCGTAG	CGGTAAGCATATTGACCAGC	77
*rpsQ*	30S ribosomal protein S17	CGTACGACTAAGATCCATGC	CCAGGGTCCAAGATTTAGTC	109
*SO2523*	TonB-dependent phytase receptor	GCCTTGATCTCTACCAAGAC	GCTATAATAGGGCTCATCGG	119
*SO2519*	Transcriptional regulator AraC family	GCAGATCCTGGAGATTAGC	CTAAAGGATAACGAGGAGGC	162
*SO3332*	Transcriptional regulator CopG family	ACCCTCTATGCAATGGGAGA	TCCACGGGGAATTCTATCAC	80
*gyrA*^a^	DNA gyrase subunit A	CGTATCGATGAGATCCAAGG	CATCCTCTTCCTCTAAAGGC	121
*gyrB*^a^	DNA gyrase B subunit B	GATGGTGGTACTCACTTAGC	GAGCTGAACTTAGGATCAGG	173

^a^Reference genes for gene expression normalization.

All primers were designed for the present study.

## Results and discussion

In the present study we evaluated the response of *S*. *oneidensis* to the deprivation of background radiation during early- and late-exponential growth phases. Liquid cultures of *S*. *oneidensis* did not show significant differences in optical density between the two treatments over the course of the experiment, as previously reported [17]. However, because it has been previously observed that cell density as an endpoint to measure the effect of our treatment lacks the resolution needed to detect such a subtle change in environmental growth conditions, we performed RNASeq-based transcriptome analysis to explore the genome-wide response in our experiment. For this purpose, a total of 12 Illumina libraries (6 early- and 6 late-exponential) were sequenced as 50 bp reads. Principal components analysis identified one of the late-exponential control libraries as an outlier (S1 Fig). When we examined the kinetics of the growth of one of the cultures that were used in this PCA data point, it was found that the culture in question had prematurely entered stationary phase, and so from these two analyses, it was decided to exclude this library from downstream processing (S1 Fig).

**Fig 2:**
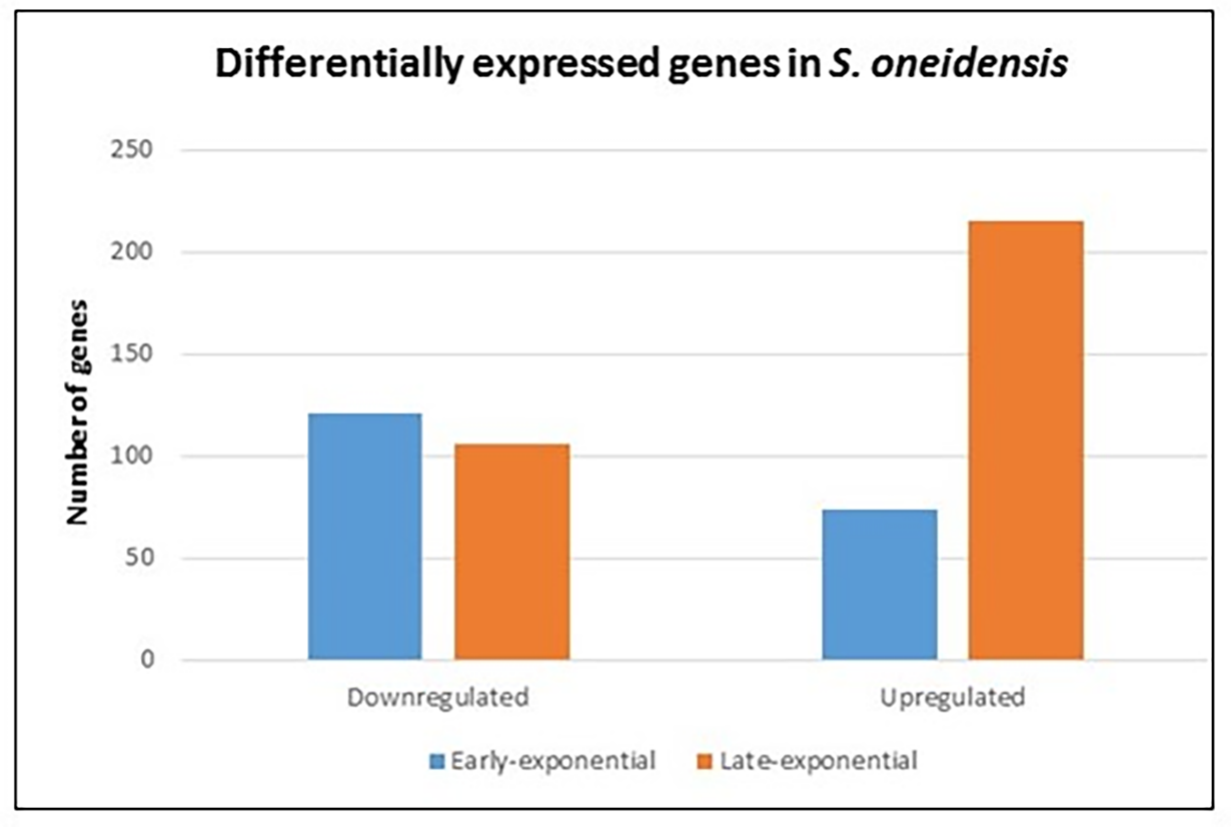
Gene regulation summary. Only genes with an FDR value ≤ 0.1 and a log_2_ differential expression ≥ 2 were considered significantly regulated.

**Fig 3:**
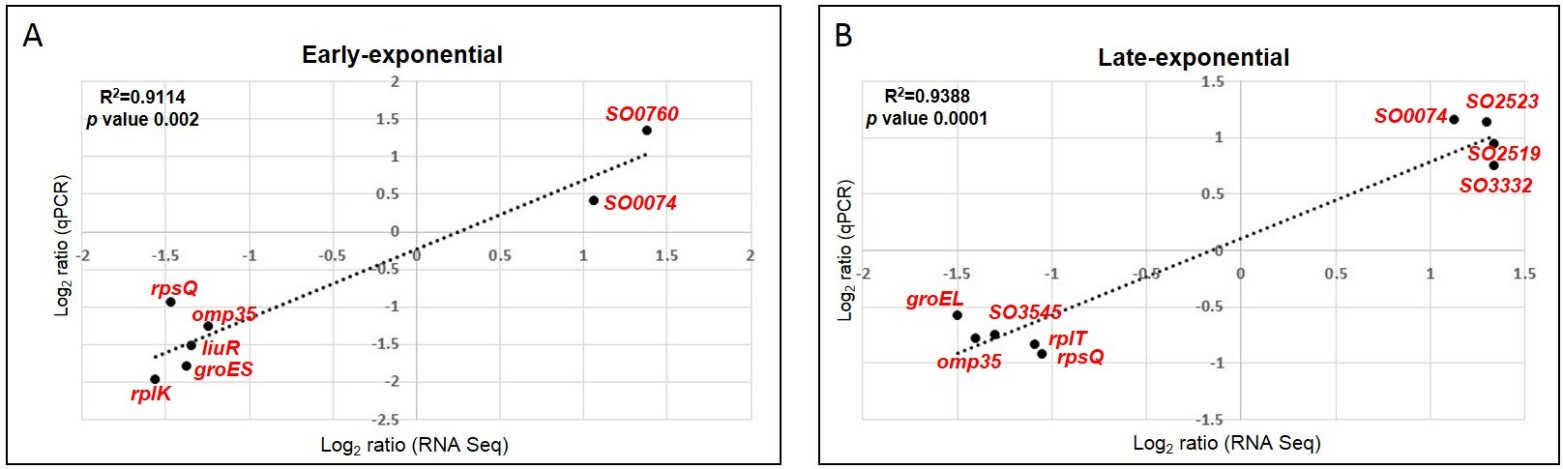
Transcriptome qPCR validation. Correlation of RNASeq and qPCR relative expression of selected genes for early (A) and late-exponential (B) transcriptomes used for transcriptome validation. Relative expression with qPCR was normalized with *gyrA* and *gyrB* as reference genes using the amplification efficiency-corrected model by Pfaffl et al. [27].

On average, the libraries had between 14,735,800 and 16,791,556 million reads, of which 83.96 to 95.5% were uniquely mapped to the *S*. *oneidensis* reference genome (NC004347, NC004349; Table 2). Only genes with a *p-adj*≤ 0.1 and a log_2_ ratio ≥ 2 were deemed significant and used for posterior analyses (S1 Table and Table 3). Using background and below-background libraries as control and treatment, respectively, we identified a total of 194 and 320 regulated genes as a result of shielding cells from background radiation during early and late stationary phase, respectively. These numbers represent the 4.6% and 7.6% of the total genes in the *S*. *oneidensis* genome, within the range of other studies with the same species that report regulations of 1.5% [28], 14% [21], and 21% [29] under different types of stress. The number of downregulated genes decreased from 120 during early- to 105 during late-stationary, whereas the number of upregulated genes increased from 74 during early- to 215 during late-stationary (Fig 2). The validity of our transcriptome analysis is shown by the significant correlation (*R*^*2*^ = 0.93) of a subset of genes amplified with RT-qPCR and compared to the expression levels obtained with RNASeq (Fig 3).

**Table 2:** RNA libraries’ statistics.

	Early stationary		Late stationary	
	Control	Treatment	Control	Treatment
Total mapped reads*	14735800 ± 2922739	15707726 ± 462826	16325039 ± 1687927	16791556 ± 1093801
Uniquely mapped reads	14082585 ± 2852924	14959442 ± 335500	13617301 ± 317119	15463545 ± 849627
% uniquely mapped reads	95.5 ± 0.475	95.25 ± 0.76	83.96 ± 10.62	92.13 ± 1.46

*Number of reads are the mean of three libraries generated from three biological replicates.

**Table 3:** Selected differentially expressed genes during early and late-exponential phases of *S*. *oneidensis* cultures. Only genes with an FDR value ≤ 0.1 and a log_2_ differential expression ≥ 2 are shown.

Gene ID	Gene name	Gene function	Expression (Log_2_)	
			Early	Late
**Translation**				
**SO0220**	*rplK*	Ribosomal protein L11	-1.56	
**SO0221**	*rplA*	Ribosomal protein L1	-1.38	
**SO0223**	*rplL*	Ribosomal protein L7/L12		-1.34
**SO0226**	*rpsL*	Ribosomal protein S12	-1.20	
**SO0227**	*rpsG*	Ribosomal protein S7	-1.08	
**SO0229**	*tufA*	Elongation factor Tu 2		-1.01
**SO0233**	*rplW*	Ribosomal protein L23	-1.22	
**SO0234**	*rplB*	Ribosomal protein L2	-1.07	
**SO0236**	*rplV*	Ribosomal protein L22		-1.07
**SO0237**	*rpsC*	Ribosomal protein S3		-1.03
**SO0238**	*rplP*	Ribosomal protein L16		-1.12
**SO0239**	*rpmC*	Ribosomal protein L29	-1.45	
**SO0240**	*rpsQ*	Ribosomal protein S17	-1.47	-1.06
**SO0241**	*rplN*	Ribosomal protein L14	-1.24	
**SO0244**	*rpsN*	Ribosomal protein S14		-1.09
**SO0247**	*rplR*	Ribosomal protein L18	-1.20	
**SO0248**	*rpsE*	Ribosomal protein S5	-1.04	
**SO0249**	*rpmD*	Ribosomal protein L30		-1.13
**SO0250**	*rplO*	Ribosomal protein L15	-1.15	
**SO0252**	*rpmJ*	Ribosomal protein L36	-1.01	-1.07
**SO0253**	*rpsM*	Ribosomal protein S13		-1.02
**SO0257**	*rplQ*	Ribosomal protein L17p	-1.04	-1.51
**SO0513**	*yaeJ*	Peptidyl-tRNA hydrolyzing factor		1.09
**SO0604**	*hflX*	GTP-binding protein	-1.04	
**SO1207**	*rpsO*	Ribosomal protein S15		-1.08
**SO1357**	*rpsP*	Ribosomal protein S16	-1.25	
**SO1629**	*rpsB*	Ribosomal protein S2	-1.18	
**SO2112**	*rplY*	Ribosomal protein L25	-1.45	
**SO2302**	*rplT*	Ribosomal protein L20	-1.59	-1.09
**SO2328**	*efp*	Translation elongation factor P	-1.06	
**SO2402**	*rpsA*	Ribosomal protein S1		-1.10
**SO3403**	*raiA*	Ribosome-associated inhibitor A		-1.31
**SO3422**	*yfiA*	Ribosomal associated cold shock response protein		-2.11
**SO3652**	*rplU*	Ribosomal protein L21	-1.34	
**SO3927**	*rplI*	Ribosomal protein L9	-1.56	
**SO3928**	*rpsR*	Ribosomal protein S18	-1.16	
**SO3939**	*rpsI*	Ribosomal protein S9	-1.35	
**SO3940**	*rplM*	Ribosomal protein L13	-1.09	
**SOt002**	*tRNA-Sec*	tRNA-Sec-1	-9.39	
**SOt003**	*tRNA-Ile-1*	tRNA-Ile-1	-1.06	
**SOt004**	*tRNA-Ala-1*	tRNA-Ala-1	-1.22	
**SOt005**	*tRNA-Thr-2*	tRNA-Thr-2	-1.08	-1.04
**SOt007**	*tRNA-Gly-6*	tRNA-Gly-6		-1.20
**SOt008**	*tRNA-Thr-1*	tRNA-Thr-1		-1.12
**SOt009**	*tRNA-Gly-4*	tRNA-Gly-4	-1.17	
**SOt010**	*tRNA-Gly-3*	tRNA-Gly-3	-1.11	
**SOt012**	*tRNA-Gly-1*	tRNA-Gly-1	-1.03	
**SOt014**	*tRNA-Met-8*	tRNA-Met-8	-1.19	
**SOt022**	*tRNA-Met-6*	tRNA-Met-6	-1.22	
**SOt024**	*tRNA-Pro-1*	tRNA-Pro-1	-1.14	
**SOt036**	*tRNA-Leu-1*	tRNA-Leu-1	-1.51	
**SOt043**	*tRNA-Leu-6*	tRNA-Leu-6	-1.45	
**SOt045**	*tRNA-Tyr-4*	tRNA-Tyr-4	-1.48	
**SOt046**	*tRNA-Tyr-3*	tRNA-Tyr-3	-1.33	
**SOt047**	*tRNA-Tyr-2*	tRNA-Tyr-2	-1.19	
**SOt055**	*tRNA-Lys-7*	tRNA-Lys-7	-1.03	
**SOt060**	*tRNA-Lys*	tRNA-Lys-8	-1.01	
**SOt063**	*tRNA-Ala-5*	tRNA-Ala-5	-1.22	
**SOt064**	*tRNA-Ile-3*	tRNA-Ile-3	-1.06	
**SOt065**	*tRNA-Glu-6*	tRNA-Glu-6	-1.39	
**SOt066**	*tRNA-Glu-5*	tRNA-Glu-5	-1.24	
**SOt067**	*tRNA-Glu-4*	tRNA-Glu-4	-1.26	
**SOt068**	*tRNA-Glu-3*	tRNA-Glu-3	-1.28	
**SOt069**	*tRNA-Glu-2*	tRNA-Glu-2	-1.37	
**SOt072**	*tRNA-Ala-3*	tRNA-Ala-3	-1.31	
**SOt073**	*tRNA-Val-5*	tRNA-Val-5	-1.04	
**SOt074**	*tRNA-Val-4*	tRNA-Val-4	-1.06	
**SOt075**	*tRNA-Val-3*	tRNA-Val-3	-1.08	
**SOt076**	*tRNA-Val-2*	tRNA-Val-2	-1.21	
**SOt077**	*tRNA-Val-1*	tRNA-Val-1	-1.58	
**SOt083**	*tRNA-Arg-8*	tRNA-Arg-8	-1.09	
**SOt084**	*tRNA-Ser-4*	tRNA-Ser-4	-1.64	
**SOt087**	*tRNA-Arg-5*	tRNA-Arg-5	-1.11	
**SOt088**	*tRNA-Arg-4*	tRNA-Arg-4	-1.14	
**SOt089**	*tRNA-Arg-3*	tRNA-Arg-3	-1.03	
**SOt090**	*tRNA-Ser-3*	tRNA-Ser-3	-1.65	
**SOt094**	*tRNA-Leu-3*	tRNA-Leu-3		-1.1764
**SOt095**	*tRNA-Ala-2*	tRNA-Ala-2	-1.22	
**SOt096**	*tRNA-Ile-2*	tRNA-Ile-2	-1.06	
**SOt097**	*tRNA-Pro-3*	tRNA-Pro-3	-1.05	
**SOt098**	*tRNA-Pro-2*	tRNA-Pro-2	-1.15	
**SOt100**	*tRNA-Arg-2*	tRNA-Arg-2	-1.01	
**SO0393**	*fis*	DNA-binding protein		-1.15
**SO3579**	*rluD*	Pseudouridine synthase		-1.03
**Chaperones**				
**SO0703***groES*10 kDa chaperonin-1.37–1.16				
**SO0704***groEL*60 kDa chaperonin-1.49				
**SO1126***dnaK*Chaperone-1.29				
**Biosynthesis of amino acids**				
**SO0276**	*argB*	Acetylglutamate kinase		1.00
**SO0279**	*argH*	Argininosuccinate lyase		1.10
**SO0818**	metE	Homocysteine methyltransferase	1.13	1.32
**SO1361**	*aroF*	Phospho-2-dehydro-3-deoxyheptonate aldolase		1.01
**SO1770**	garK	Glycerate kinase	1.01	
**SO2074**	hisG	ATP phosphoribosyltransferase	1.11	1.20
**SO2767**	asnB	Asparagine synthetase [glutamine-hydrolyzing]	1.02	
**SO3986**	*lysC*	Aspartokinase		1.11
**SO4245**	argA	N-acetylglutamate synthase	1.01	1.11
**SO2305**	*lrp*	Leucine-responsive regulatory protein	-1.10	
**SO1898**	*liuR*	Transcriptional regulator LiuR of Leu degradation	-1.35	
**ABC transporters**				
**SO0056**	* *	Transport system substrate binding component		1.05
**SO0070**	*natA*	ABC-type sodium efflux system ATPase component		1.04
**SO0073**	* *	ABC-type efflux system ATPase component		1.11
**SO0074**		ABC transporter, permease protein	1.06	1.14
**SO0525**	rmrB	Multidrug resistance protein	1.05	1.36
**SO0821**	macB	Macrolide export ATP-binding/permease protein	1.03	
**SO0822**	macC	RND efflux system, outer membrane lipoprotein	1.01	1.07
**SO0858**	*glyP*	Na(+)-linked D-alanine glycine transporter		1.05
**SO1034**	*btuC*	Cobalamin uptake system permease component		1.08
**SO1273**	potI	Putrescine transport system permease protein	1.36	1.25
**SO1647**	*kefB*	Glutathione-gated K(+)-efflux system		1.07
**SO1760**		AzlC family protein	1.46	
**SO1918**	* *	Multidrug and toxin efflux protein MATE family		1.06
**SO3485**	*emrD3*	Multidrug efflux pump		1.03
**SO3674**	hmuC	Hemin ABC transporter, permease protein	1.17	
**SO3691**	* *	Macrolide export system permease component 2		1.20
**SO3692**	* *	Macrolide export system ATPase component		1.02
**SO3694**		ABC transporter, permease protein	1.03	1.50
**SO4447**	modB	Molybdenum transport system permease protein	1.24	1.01
**SO4527**		Permease of the drug/metabolite	1.11	1.21
**Other transporters**				
**SO0057**	*ktrB*	Na-dependent K uptake membrane component		1.25
**SO0058**	*ktrA*	Na-dependent K uptake NAD binding component		1.15
**SO0157**	* *	Proton:glutamate symporter DAACS family		1.24
**SO0194**	* *	Acyl transferase		1.17
**SO0455**	* *	alpha-ketoglutarate uptake system		1.01
**SO0715**	*sorA*	SO3 dehydrogenase molybdopterin-binding subunit		1.05
**SO0737**	*nicT*	TonB-dependent nickel receptor		1.18
**SO0760**	amt	Ammonium transporter	1.38	1.42
**SO1047**	lrgA	Holin-like protein CidA	1.23	
**SO1917**	* *	Major facilitator superfamily transporter		1.07
**SO2195**	* *	Inter-alpha-trypsin inhibitor family protein		1.31
**SO2523**		TonB-dependent receptor	1.05	1.31
**SO2713**	pnuT	Predicted thiamin transporter	1.15	1.13
**SO3503**	nagP	N-acetyl glucosamine transporter	1.07	
**SO4004**	* *	Proton/sodium:glutamate symporter DAACS family		1.03
**SO4050**	* *	Putative transport system permease component		1.12
**SO4081**	*puuP*	Putrescine uptake protein PuuP		1.14
**SO4296**	*nupC*	Na-dependent nicotinamide ribose transporter		1.20
**SO4339**	* *	Transporter		1.24
**Respiration**				
**SO0259**	*ccmE*	Cytochrome c-type biogenesis protein		-1.26
**SO0260**	*ccmD*	Heme export system CcmE-interacting component		-1.32
**SO0261**	*ccmC*	ABC-type heme export system permease component 2		-1.10
**SO0264**	*scyA*	Cytochrome c-type protein	-1.41	
**SO0476**	*sirH*	Cytochrome c maturation periplasmic thioredoxin		1.09
**SO0477**	sirF	Cytochrome c maturation system	1.10	1.21
**SO0478**	*sirE*	Cytochrome c maturation system haem lyase subunit		1.36
**SO0479**	*sirA*	Sulfite reductase SirA		1.10
**SO0480**	*sirB*	Sulfurtransferase SirB		1.20
**SO0481**	*sirI*	Peptidyl-prolyl cis-trans isomerase		1.03
**SO0483**	*sirC*	4Fe-4S ferredoxin SirC		1.09
**SO0484**	*sirD*	Menaquinol oxidase		1.15
**SO0485**	*nosL*	Copper uptake periplasmic chaperone component		1.00
**SO0488**	*nosY*	C copper transport system permease component		1.08
**SO0630**	*nosA*	TonB-dependent copper receptor		1.01
**SO0714**	* *	Periplasmic monoheme cytochrome c4		1.14
**SO0717**	* *	Periplasmic monoheme cytochrome c4		1.55
**SO0845**	napB	Nitrate reductase cytochrome c550-type subunit	1.28	
**SO0846**	napH	Polyferredoxin NapH (periplasmic nitrate reductase)	1.43	
**SO0848**	napA	Periplasmic nitrate reductase precursor	1.11	1.13
**SO0849**	*napD*	Periplasmic nitrate reductase chaperone		1.12
**SO0904**	*nqrC*	Na(+)-translocating NADH-quinone reductase subunit C		-1.09
**SO0970**	*fccA*	periplasmic fumarate reductase	-1.43	-1.97
**SO1233**	torC	TMAO reductase associated c-type cytochrome	1.32	1.33
**SO1413**	* *	Flavocytochrome c heme submit		1.27
**SO1414**	* *	Flavocytochrome c flavin subunit		1.29
**SO1776**	*mtrB*	FeO respiratory outer membrane component		-1.36
**SO1777**	*mtrA*	FeO respiratory cytochrome c component		-1.51
**SO1778**	*mtrC*	FeO respiratory se cytochrome c component		-1.49
**SO1779**	*omcA*	D decaheme cytochrome c lipoprotein	-1.04	-1.72
**SO1929**	*sdhB*	Succinate dehydrogenase iron-sulfur protein	-1.02	
**SO2361**	*ccoP*	Cbb3-type cytochrome c oxidase subunit		-1.06
**SO2362**	*ccoQ*	Cytochrome c oxidase (cbb3-type) subunit CcoQ	-1.18	-1.20
**SO2931**	* *	Cytochrome c lipoprotein		1.20
**SO3058**		Flavocytochrome c flavin subunit	1.14	1.05
**SO3286**	*cydA*	Cytochrome d ubiquinol oxidase subunit I		-1.01
**SO3325**	*nrfJ*	Uncharacterized protein		-1.38
**SO3885**		AAA ATPase, central domain protein	1.02	
**SO4142**	* *	Periplasmic monoheme cytochrome c		1.29
**SO4144**	*otr*	Octaheme tetrathionate reductase		1.17
**SO4202**	*tatA*	Twin-arginine translocation protein	-1.13	
**SO4483**	* *	Cytochrome b		1.14
**SO4484**	*shp*	Monoheme cytochrome c		1.13
**SO4568**	*nrfD*	Nitrite reductase quinol dehydrogenase component		1.50
**SO4591**	*cymA*	Cytochrome c-type protein		-1.45
**SO4607**	*coxA*	Aa3 type cytochrome c oxidase subunit I		1.03
**SO4608**	ctaG	Cytochrome oxidase biogenesis protein	1.05	
**SO4614**	*ctaB*	Protoheme IX farnesyltransferase		1.10
**SO4694**	*torF*	TMAO reductase system outer membrane porin		1.38
**SO4746**	*atpC*	ATP synthase epsilon chain	-1.16	
**SOm003**	* *	tmRNA	-1.50	
**Transcriptional regulators**				
**SO0624**	*crp*	cAMP-responsive regulator of catabolite repression		-1.33
**Porins**				
**SO3896**	*omp35*	Outer membrane porin, putative	-1.24	-1.40
**SO3545**	*ompW*	Outer membrane protein		-1.37
**SO2194**	*ompA*	Outer membrane porin		-1.30

### Gene ontology (GO) term enrichment analysis

**Fig 4:**
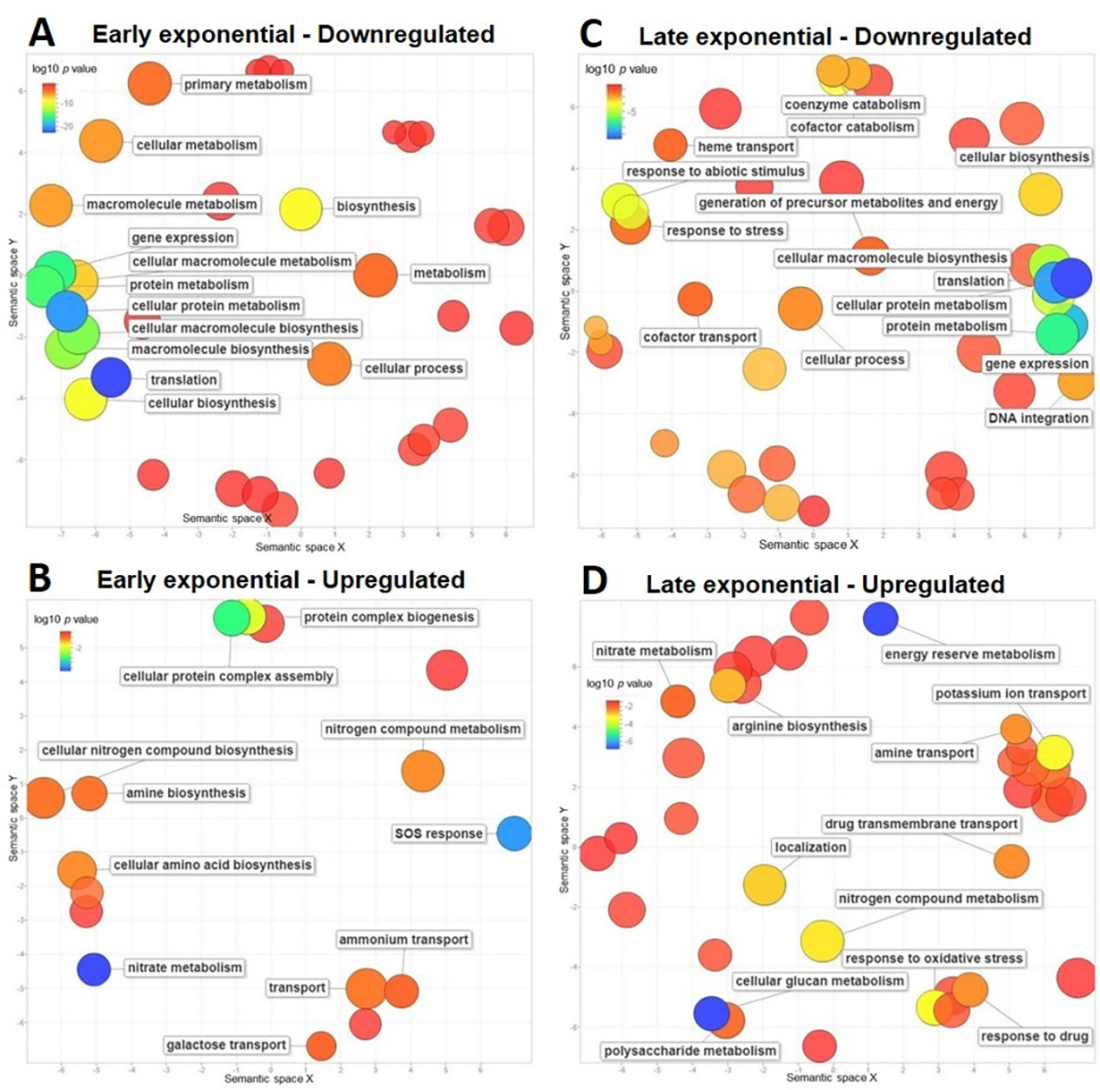
Biological process GO terms enrichment analysis in response to below-background radiation in *S*. *oneidensis*. GO terms redundancy in down and upregulated genes was reduced and summarized using REVIGO [26]. Scatterplots show the non-redundant, down and upregulated GO terms enriched during early (A, B) and late (C, D) exponential phases. Only GO terms with a log10 *p* value ≤ -1.5 are labeled and the bubble size indicates the frequency of the GO term.

**Fig 5:**
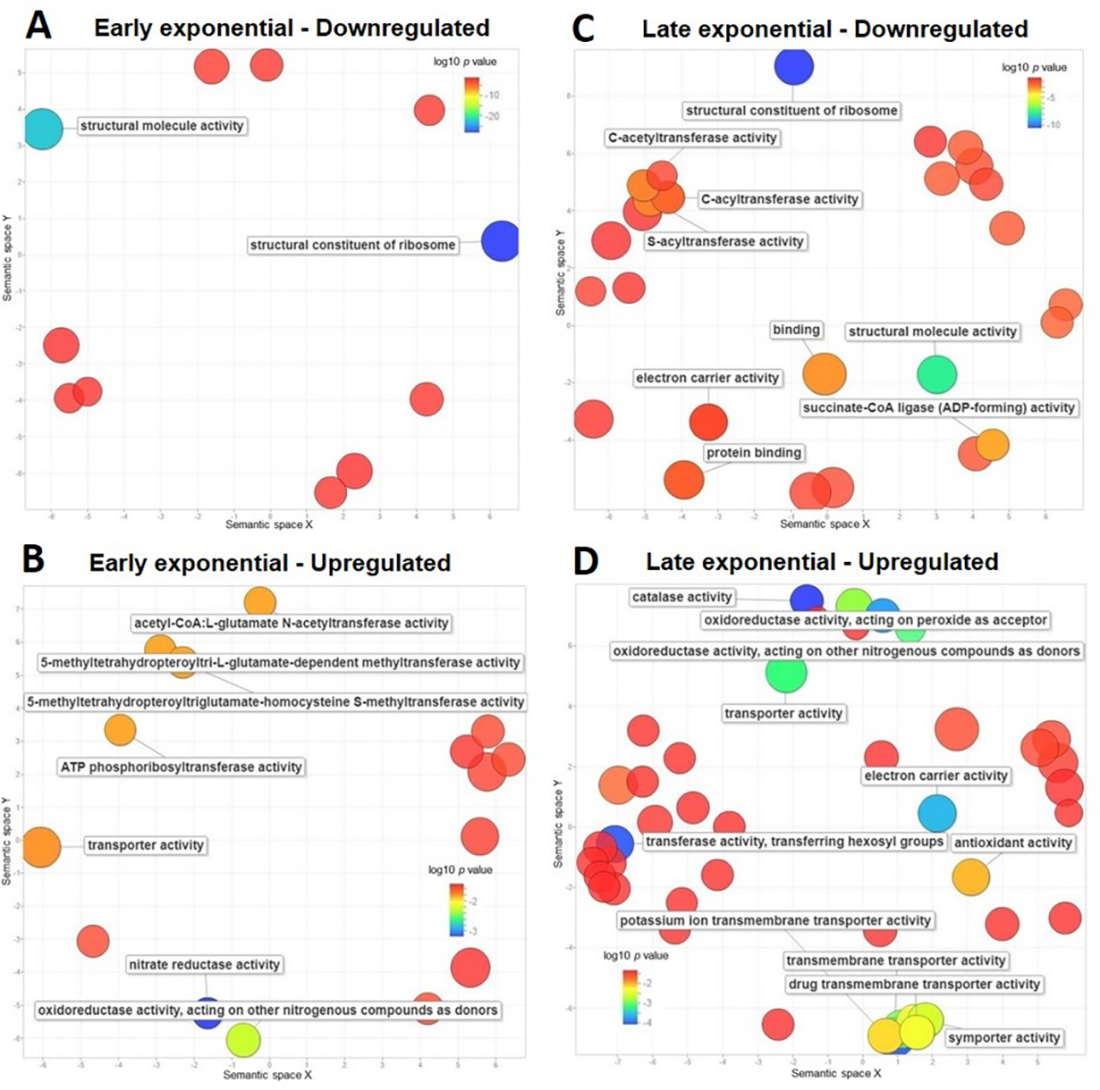
Metabolic function GO terms enrichment analysis in response to below-background radiation in *S*. *oneidensis*. GO terms redundancy in down and upregulated genes was reduced and summarized using REVIGO [26]. Scatterplots show the non-redundant, down and upregulated GO terms enriched during early (A, B) and late (C, D) exponential phases. Only GO terms with a log10 *p* value ≤ -1.7 are labeled and the bubble size indicates the frequency of the GO term.

GO term enrichment was performed to group the differentially regulated genes according to their function. In the radiation-shielded treatment, both growth phases were characterized by the dominant downregulation of the processes: translation (GO:0006412), protein metabolism (GO:0019538), and gene expression (GO:0010467). Similarly, the GO terms nitrogen metabolic process (GO:0006807) and transport (GO:0006810) were upregulated during both phases; SOS response (GO:0009432) was upregulated during early-exponential and both cellular glucan metabolic process (GO:0006073) and energy reserve metabolic process (GO:0006112) during late-exponential phase (Fig 4). Metabolically, the terms structural constituent of the ribosome (GO:0003735) and structural molecule activity (GO:0005198) were significantly downregulated in both phases (Fig 5). Comparatively, the terms active transmembrane transporter activity (GO:0022804) and transporter activity (GO:0005215) were upregulated during both phases, while ATPase activity (GO:0016887) was upregulated during early-exponential and electron carrier activity (GO:0009055) and antioxidant activity (GO:0016209) were upregulated during late-exponential (Fig 5).

### Ribosomal proteins, tRNAs and translation factors

**Fig 6:**
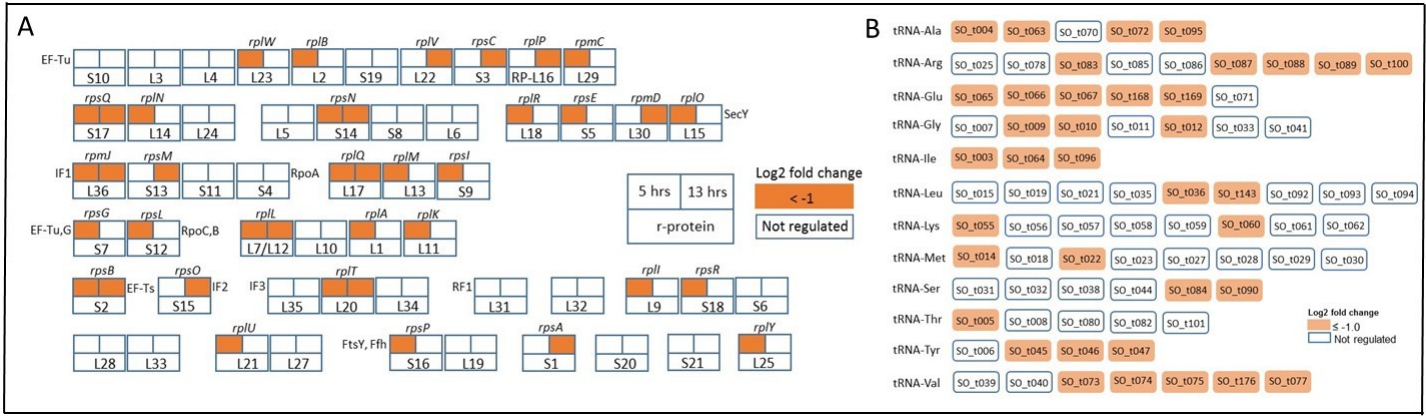
**Overview of the downregulation of *S*. *oneidensis* (A) ribosomal proteins during early- and late-exponential, and (B) tRNA genes during early-exponential phases.** Adapted from the KEGG ribosomal proteins pathway, panel A shows the downregulation of each ribosomal protein in either early (upper left corner), late-exponential (upper right corner) or both phases.

The expression pattern in our experiments shows that the deprivation of background levels of radiation caused a marked downregulation of a significant number of ribosomal protein-coding genes during both early- (21% of all annotated ribosomal proteins) and late-exponential (14%) phase cultures (Fig 6A). These data suggest that an important initial response to radiation deprivation is to slow protein synthesis by reducing the number of translationally active ribosomes. Ribosomal proteins play a wide variety of roles in ribosomes, and so a decrease in their expression levels has the potential to disrupt optimal translational activity as well. For example, proteins L15 (*rplO*), L16 (*rplP*) and L20 (*rplT*) contribute to the stability of the ribosomes [30]); S1 (*rps*A) participates in the peptide chain elongation [31]; S3 (*rpsC*) and S5 (*rpsE*) assist unwinding the mRNA secondary structure as it enters the A site [32]; L1 (*rplA*) releases the deacylated tRNA from the E site allowing the re-occupation of the A site [33]; L7/L12 *(rplL*) are in involved in the binding of the elongation factor by activating its GTPase activity [34] and L11 (*rplK*) senses the presence of uncharged tRNA’s in the A-site, triggering the stringent response [35], among others.

As ribosomes account for up to 40% of the *Escherichia coli* cell’s dry weight [36] and protein synthesis accounts for up to 50% of the cellular energy [37], ribosome synthesis and protein translation are two critical processes that are tightly regulated to meet the protein synthesis rate required under specific growth conditions [38]. The downregulation of ribosomal proteins in *Shewanella* has been associated with numerous stressors: Acidic stress [39], heat shock [40], exposure to a magnetic field [28], and chromate stress [29]. Ribosomal protein downregulation has been reported as well in other microbial genera in response to stress: in *Campylobacter jejuni* under elevated growth temperature [41]; in *Streptococcus pneumoniae* treated with antimicrobial peptides [42]; in *Staphylococcus aureus* under acid shock [43], in *Corynebacterium glutamicum* under suboptimal oxygen conditions [44], and in *Saccharomyces cerevisiae* exposed to the alkylating agent methyl methanesulfonate [45].

Other components of the translational machinery that were downregulated are genes coding for tRNAs. During early log phase, thirty seven tRNA genes were downregulated, none were upregulated. These genes are involved in the synthesis of twelve types of aminoacyl-tRNAs, with all (3 of 3 for Ile) or most (3 of 4 for Tyr, 4 of 5 for Ala, 5 of 6 for Glu) of the isotypes downregulated (Fig 6B). It has been reported that the tRNA-gene copy number determines the number of tRNAs involved in translation [46] and, interestingly, both conditions of oxidative stress and stringent response in *E*. *coli* elicit a similar downregulation of these genes [47, 48]. Additionally, *f*is, a growth phase-dependent regulator involved in the regulation of tRNA levels [49, 50] was also downregulated, as previously observed in *S*. *oneidensis* under acid/alkaline conditions [39].

Our experiment also revealed the regulation of other translation-related genes during both early- and late-exponential phases (Table 3). During early-exponential, we observed the upregulation of *yaeJ*, a peptidyl-tRNA hydrolyzing factor known to relieve stalled ribosomes [51, 52], and the downregulation of *efp*, a translation elongation factor responsible for the formation of the peptide bond between the first and second amino acids during translation. Later on during late-exponential phase, the reduced expression of *tufA* (elongation factor Tu), *raiA* (ribosome-associated inhibitor A), *yfiA* (ribosome-associated cold-shock response protein) and *rluD* (23S rRNA pseudouridine synthase) suggest an important reduction in ribosomal stability as a result of a lower concentration of EF-Tu to bind the aminoacyl-tRNAs to the ribosome [53, 54], a weaker stabilization of the 70S ribosome against dissociation [55], and the decreased rigidity of the 23S rRNA as a consequence of the lower expression of pseudouridine [56]. The consistent downregulation of ribosomal proteins and tRNAs, as well as the effects involving protein synthesis, all indicate a “pause” in translation, and we interpret this as a response which allows for a remobilization of amino acid and protein resources in order to re-establish homeostasis in response to the stress of reduced radiation. Similarly, the molecular chaperones *groES*, *groEL*, and *dnaK* were downregulated during late-exponential phase, adding to our hypothesis that reduced background radiation lowers the immediate need for active proteins. Although this downregulation opposes the “traditional” response of cells under stress, it has been previously observed in *S*. *oneidensis* [39] and in *E*. *coli* [57] under acidic stress and heat shock under microgravity conditions, respectively.

### Amino acids biosynthesis and metabolism

Our transcriptome analysis showed the early-exponential upregulation of genes involved in the flux of several amino acids, such as the biosynthesis of arginine (*argA*, amino acid acetyltransferase; *argB*, acetylglutamate kinase; *argH*, argininosuccinate lyase), histidine (*hisG*, phosphoribosyltransferase), lysine (*lysC*, lysine-sensitive aspartokinase III), phenylalanine, tyrosine, and tryptophan (*aroF*, phosphor-s-dehydro-deoxyheptonate aldolase tyr-sensitive) and the metabolism of cysteine and methionine (*metE*, B12-independent 5-methyltetrahydropteroyltriglutamate-homocysteine methyltransferase), alanine, asparagine, and glutamate (*asnB*, asparagine synthase glutamine-hydrolyzing), glycine, serine, and threonine (*garK*, glycerate kinase), glycine, serine, threonine, cysteine, and methionine (*lysC*, lysine-sensitive aspartokinase III), and histidine (*hisG*, ATP phosphorybosiltransferase). Also, the downregulation of the leucine-responsive regulatory protein (*lrp*) and the transcriptional repressor of branched chain amino acid degradation (*liuR*) suggest the modulation of the metabolism of one-carbon compounds, amino acids, and sugars, and the assimilation of nitrogen [58] and a lower branched chain amino acids catabolism [59], respectively. Hence, another tactic taken by *Shewanella*, probably related to the above-mentioned remobilization of proteins, is the adjustment of the biosynthesis and degradation of numerous amino acids.

### Membrane transport and cell to cell communication

Multi-drug resistance (MDR) pumps, essential for antibiotic resistance within a clinical context, also have natural roles in detoxification processes and in the maintenance of cellular homeostasis [60–62]. Notably, we identified the upregulation of ABC and other transporters-related genes that increased from 17 during early- to 30 during late-exponential phase (Table 3). Although most of these genes are involved in the efflux of macrolides and drugs (*SO1918*, *emrD*, *rmrB*, *SO4527*, *SO3691*, *macC*, *SO3694*, *SO3692*, *SO1917*), some are related to the uptake of Fe (*nicT*, *SO2523*), alpha ketoglutarate (*SO0455*), potassium (*ktrA*, *ktrB*), vitamin B12 (*btuC*), putrescine (*potI*), amino acids (*SO0056*), molybdate (*modB*), and hemin (*hmuC*). Similar induction of efflux pump genes has been observed in *S*. *oneidensis* under acidic [39] and chromate [29] stresses, and in response to UVA [20] and in *S*. *algae* under salt stress [63].

The passive movement of hydrophilic molecules through porins exerts a major control of the uptake nutrients, and potentially, signaling molecules. It has been reported that the cAMP-activated global transcriptional regulator CRP positively regulates the expression of the *ompA* gene, that codes for the structural component of porins in *E*. *coli* [64] and for *ompW* and *omp35* in *S*. *oneidensis* [65]. In our experiment, both *crp* and *ompA* genes were downregulated during late-exponential phase, along with *ompW*, whose product participates in the uptake of cations in *Caulobacter crescentus* [66], supporting our hypothesis that cell signaling is diminished upon deprivation of background levels of radiation.

### Respiration

*Shewanella oneidensis* is a facultative anaerobic bacterium capable of using a wide variety of terminal electron acceptors; its genome encodes 44 c-type cytochromes [67], and most of them function as terminal reductases [68]. The number of respiration-related processes that were regulated in our experiments increased from 16 during early- to 41 during the late-exponential phase, indicating a shift in the potential use of terminal electron acceptors as the cultures approached stationary phase in the radiation-deprived condition. At the beginning of growth, the upregulation of genes involved in oxidative phosphorylation (*ctaG*, *SO3058*, *coxA*) indicates an increase in electron-transporting activity and the hydrolysis of ATP (*SO3885*), along with the downregulation of subunits for the synthesis of a cytochrome c-oxidase (*ccoQ*), an ATPase (*atpC*), a periplasmic monoheme cytochrome C (*scyA*), and a succinate dehydrogenase (*sdhB*) responsible for the reduction of ubiquinone to ubiquinol. Towards the end of exponential growth, the upregulation of *coxA* is maintained, accompanied by the downregulation of two subunits for the cbb3-type cytochrome c-oxidase (*ccoQ*, *ccoP*), one subunit of the cytochrome bd complex (*cydA*), and three components of the cytochrome c maturation (*Ccm*) system (*ccmC*, *ccmD*, *ccmE*), responsible for the catalytic activity of cytochromes [69].

**Fig 7:**
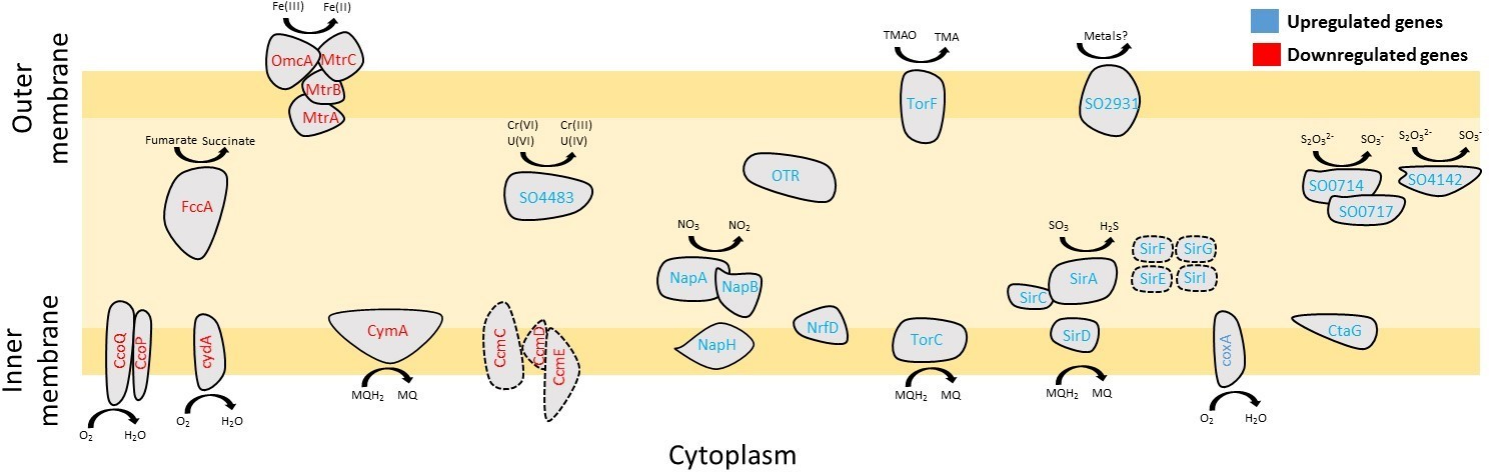
Overview of electron transport proteins genes regulated in *S*. *oneidensis* during late-exponential phase.

Our analysis also revealed the upregulation of a significant number of genes related to the use of nitrate, sulfate, TMAO, and thiosulfate as terminal electron acceptors (Fig 7). For instance, the two subunits of the nitrate reductase system (*napA*, *napB*) were upregulated throughout the experiment, suggesting the reduction of nitrate [61]. Probably related to this is the downregulation of *cymA*, which codes for a protein that transfers electrons to NapA, inhibiting the process of nitrate respiration [70]. During late-exponential additional respiratory systems were upregulated; for example, the reduction of trimethylamine oxide (*torC*, *torF*), sulfite (*sirA*, *sirE*, *sirF*, *sirG*, *sirC*, *sirD*, *sirI*, *nosY*, *nosL*), thiosulfate (*SO0714*, *SO0717*, *SO4142*), Cr(IV)/U(IV) (*SO4483*), and Fe/Mn oxides (*SO2931*). In contrast, all the components of the Mtr pathway (*mtrA*, *mtrB*, *mtrC*, *omcA*) were downregulated, suggesting a diminished ability to use membrane extension nanowire connections to use iron oxides as electron acceptors [71].

We suggest that this significant investment by *Shewanella* in increasing respiratory options and electron transport branches may be an effort on the part of the cells to compensate for the lower availability of oxidant species under the below-background radiation condition. Reactive oxygen species such as hydrogen peroxide have been shown to perform important roles in cell-cell communication [3] and gene regulation [6]. Without natural background levels of radiation, it is likely that less of these oxidants would be produced and the cell may compensate for this by increasing the generation of endogenous oxidants through alternative membrane electron transport processes.

### Chaperones and folding catalysts

Molecular chaperones, chaperonins, and heat shock proteins assist in the folding newlyformed proteins, re-folding denatured proteins into their active conformation, and prevent unfolded proteins from aggregating into non-functional states [72]. In our study, the genes coding for the major chaperone/chaperonin systems (*groES*, *groEL*, *dnaJ*, *dnaK*) and other protein folding catalysts (*clpB*, *htpG*, *secB and ibpA*) were downregulated during late-exponential phase. Such unusual pattern of downregulation of these genes has been previously observed in *S*. *oneidensis* under acidic stress [39].

## Conclusion

The present study shows the first genome-wide bacterial response, of any organism, to the extremely low levels of background and below-background radiation. In agreement with our previous work [17], the growth of *S*. *oneidensis* was not inhibited by radiation shielding. However, the regulation of different gene families, most remarkably those involved in protein translation activity, suggests that *S*. *oneidensis* cells “sensed” a change in their physical environment and responded to it by regulating their translation rate. We have also previously observed the upregulation of genes associated with oxidative stress response and DNA damage repair that suggest a stress response [16, 17]. This time, unconstrained by the limitations of targeting specific genes, we identified the regulation of a wider variety of genes involved in different metabolic processes, suggesting that exposure to some minimum amount of ionizing radiation might be required by *S*. *oneidensis* to retain homeostasis. Our hypothesis is in apparent contradiction to recently published modeling studies that suggest the improbability that the dose rates for our study could exert any effect on our cells to significantly alter their physiology [73, 74]. However, the paradigm shift we present is that the genome-wide gene regulation response in our model is precisely due to a lower intracellular radiolysis products concentration because of a reduced hit rate by radiation tracks. As we mentioned before, some ROS are catalysts for transcriptional regulation [7], therefore background-shielded cells might experience a slower transcription rate compared to their counterpart grown in the presence of background radiation. Our data suggests a decreased ability of cells to exchange substrates across the membrane, including diffusible components that might act as signaling factors. Comparatively, the bystander effect explains a population-wide effect even when only a fraction of the cells has been hit by a radiation track, precisely due to the transmission of chemical signals [75]. Interestingly, a small number of genes (twenty) regulated upon deprivation of background levels of radiation coincided with those regulated in our model when challenged with acute doses of either UV, solar (SR) or ionizing radiation (IR). For instance, *katB* expression was also induced by 558 J m^-2^ [21] and 40 Gy [19] of solar and ionizing radiation, respectively, and *umuC* and *umuD* were also upregulated by IR and 25, 568 and 3.3 J m^-2^ of UVABC [20]. The expression of these genes under such disparate conditions supports our proposal of below background radiation as a source of stress for *Shewanella* cells. In comparison, we have observed *D*. *radiodurans* growth inhibition while it only upregulated two of the five genes regulated in *S*. *oneidensis* which could be interpreted as its inability to perceive the lack of radiation as an important environmental cue [17]. Consistent with this proposal, there was a genome-wide transcriptome level response in *Shewanella* but the transcriptome response of *Deinococcus* appears much diminished (Castillo et al., in preparation), lending credence to the proposed connection between the inability to detect stress and the inability to take measures to preserve homeostasis.

Since the removal of normal levels of background radiation causes stress in bacteria (this and previous reports, [16, 17]), protozoa [10, 13], yeast [11], and mammalian cells [12–14], natural levels of radiation have, evidently, an important fitness role in biology. The identity of such a fitness role of low-level radiation and the associated mechanisms, however, are still largely unknown and will be an important research component in understanding the biological effects of low-dose radiation.

## Supporting information

S1 Fig Principal components analysis (PCA) showing RNA libraries distribution. Control and treatment libraries refer to *S*. *oneidensis* cultures grown at background and below-background doses of radiation, respectively.
(TIF)

S1 Table Regulated genes in S. *oneidensis* grown deprived from background radiation. Only genes with a FDR < 0.1 and log_2_ >1 or <-1 are shown.
(DOCX)
