# Supplementary figures and images for: Transcriptome analysis reveals a stress response of Shewanella oneidensis deprived of background levels of ionizing radiation
Source: PLoS One. 2018 May 16;13(5):e0196472. doi: 10.1371/journal.pone.0196472 (PMC5955497; doi:10.1371/journal.pone.0196472)

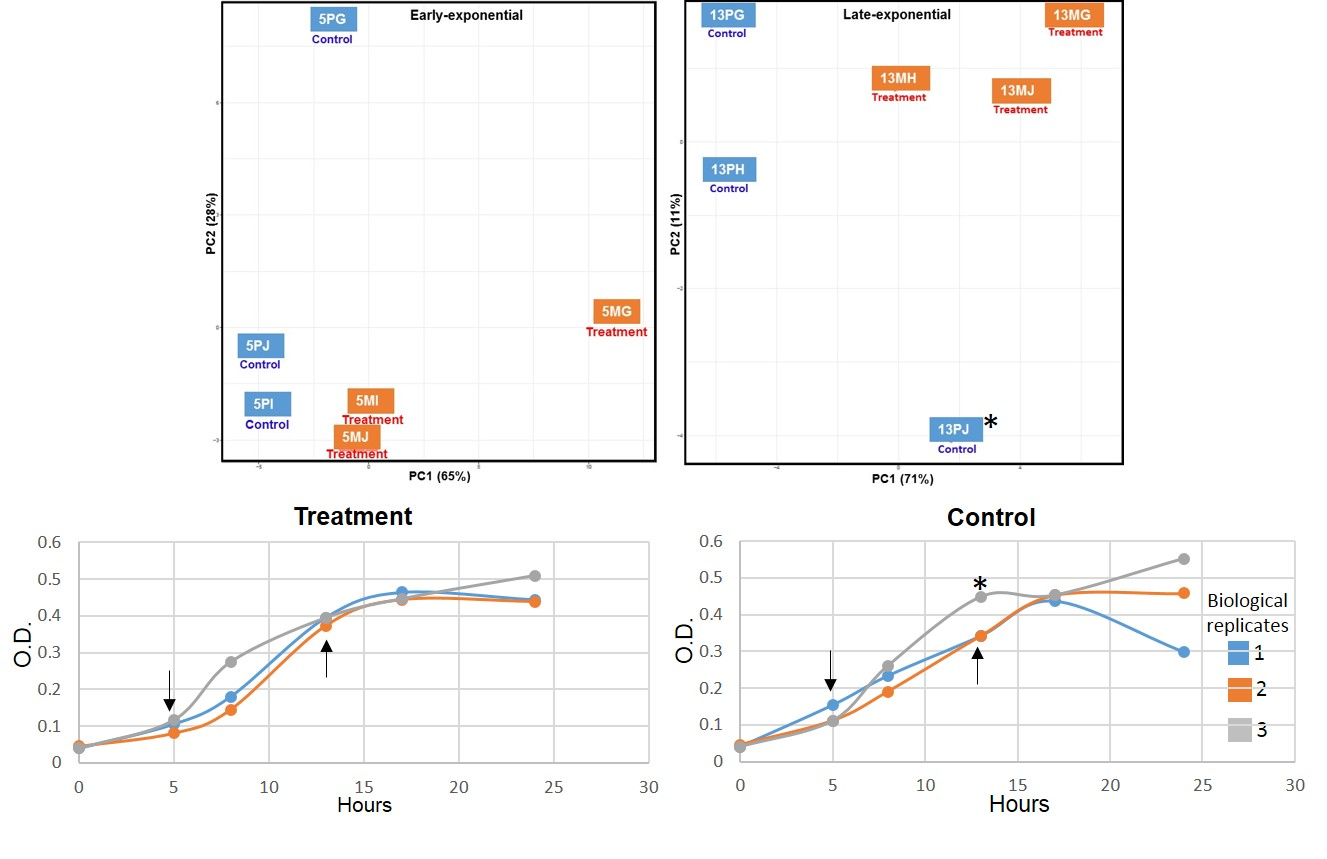

Supplement: S1 Fig — Control and treatment libraries refer to S. oneidensis cultures grown at background and below-background doses of radiation, respectively. (TIF) [file pone.0196472.s001.tif]
