# Supplementary material for: Transcriptome analysis reveals a stress response of Shewanella oneidensis deprived of background levels of ionizing radiation
Source: PLoS One. 2018 May 16;13(5):e0196472. doi: 10.1371/journal.pone.0196472 (PMC5955497; doi:10.1371/journal.pone.0196472)
Supplement: S1 Table — Only genes with a FDR < 0.1 and log2 >1 or <-1 are shown. (DOCX) [file pone.0196472.s002.docx]

**S1 Table. Regulated genes in *S. oneidensis* grown deprived from background radiation.** Only genes with a FDR < 0.1 and log2 >1 or -1 are shown.

| Gene ID | Gene name | Gene function | Expression (Log2) | |
| --- | --- | --- | --- | --- |
|  |  |  | **Early** | **Late** |
| Protein biosynthesis | | | | |
| SO0220 | *rplK* | Ribosomal protein L11 | -1.56 |  |
| SO0221 | *rplA* | Ribosomal protein L1 | -1.38 |  |
| SO0223 | *rplL* | Ribosomal protein L7/L12 |  | -1.34 |
| SO0226 | *rpsL* | Ribosomal protein S12 | -1.20 |  |
| SO0227 | *rpsG* | Ribosomal protein S7 | -1.08 |  |
| SO0229 | *tufA* | Elongation factor Tu 2 |  | -1.01 |
| SO0233 | *rplW* | Ribosomal protein L23 | -1.22 |  |
| SO0234 | *rplB* | Ribosomal protein L2 | -1.07 |  |
| SO0236 | *rplV* | Ribosomal protein L22 |  | -1.07 |
| SO0237 | *rpsC* | Ribosomal protein S3 |  | -1.03 |
| SO0238 | *rplP* | Ribosomal protein L16 |  | -1.12 |
| SO0239 | *rpmC* | Ribosomal protein L29 | -1.45 |  |
| SO0240 | *rpsQ* | Ribosomal protein S17 | -1.47 | -1.06 |
| SO0241 | *rplN* | Ribosomal protein L14 | -1.24 |  |
| SO0244 | *rpsN* | Ribosomal protein S14 |  | -1.09 |
| SO0247 | *rplR* | Ribosomal protein L18 | -1.20 |  |
| SO0248 | *rpsE* | Ribosomal protein S5 | -1.04 |  |
| SO0249 | *rpmD* | Ribosomal protein L30 |  | -1.13 |
| SO0250 | *rplO* | Ribosomal protein L15 | -1.15 |  |
| SO0252 | *rpmJ* | Ribosomal protein L36 | -1.01 | -1.07 |
| SO0253 | *rpsM* | Ribosomal protein S13 |  | -1.02 |
| SO0257 | *rplQ* | Ribosomal protein L17p | -1.04 | -1.51 |
| SO0513 | *yaeJ* | Peptidyl-tRNA hydrolyzing factor |  | 1.09 |
| SO0604 | *hflX* | GTP-binding protein | -1.04 |  |
| SO1207 | *rpsO* | Ribosomal protein S15 |  | -1.08 |
| SO1357 | *rpsP* | Ribosomal protein S16 | -1.25 |  |
| SO1629 | *rpsB* | Ribosomal protein S2 | -1.18 |  |
| SO2112 | *rplY* | Ribosomal protein L25 | -1.45 |  |
| SO2302 | *rplT* | Ribosomal protein L20 | -1.59 | -1.09 |
| SO2328 | *efp* | Translation elongation factor P | -1.06 |  |
| SO2402 | *rpsA* | Ribosomal protein S1 |  | -1.10 |
| SO3403 | *raiA* | Ribosome-associated inhibitor A |  | -1.31 |
| SO3422 | *yfiA* | Ribosomal associated cold shock response protein |  | -2.11 |
| SO3652 | *rplU* | Ribosomal protein L21 | -1.34 |  |
| SO3927 | *rplI* | Ribosomal protein L9 | -1.56 |  |
| SO3928 | *rpsR* | Ribosomal protein S18 | -1.16 |  |
| SO3939 | *rpsI* | Ribosomal protein S9 | -1.35 |  |
| SO3940 | *rplM* | Ribosomal protein L13 | -1.09 |  |
| SOt002 | *tRNA-Sec* | tRNA-Sec-1 | -9.39 |  |
| SOt003 | *tRNA-Ile-1* | tRNA-Ile-1 | -1.06 |  |
| SOt004 | *tRNA-Ala-1* | tRNA-Ala-1 | -1.22 |  |
| SOt005 | *tRNA-Thr-2* | tRNA-Thr-2 | -1.08 | -1.04 |
| SOt007 | *tRNA-Gly-6* | tRNA-Gly-6 |  | -1.20 |
| SOt008 | *tRNA-Thr-1* | tRNA-Thr-1 |  | -1.12 |
| SOt009 | *tRNA-Gly-4* | tRNA-Gly-4 | -1.17 |  |
| SOt010 | *tRNA-Gly-3* | tRNA-Gly-3 | -1.11 |  |
| SOt012 | *tRNA-Gly-1* | tRNA-Gly-1 | -1.03 |  |
| SOt014 | *tRNA-Met-8* | tRNA-Met-8 | -1.19 |  |
| SOt022 | *tRNA-Met-6* | tRNA-Met-6 | -1.22 |  |
| SOt024 | *tRNA-Pro-1* | tRNA-Pro-1 | -1.14 |  |
| SOt036 | *tRNA-Leu-1* | tRNA-Leu-1 | -1.51 |  |
| SOt043 | *tRNA-Leu-6* | tRNA-Leu-6 | -1.45 |  |
| SOt045 | *tRNA-Tyr-4* | tRNA-Tyr-4 | -1.48 |  |
| SOt046 | *tRNA-Tyr-3* | tRNA-Tyr-3 | -1.33 |  |
| SOt047 | *tRNA-Tyr-2* | tRNA-Tyr-2 | -1.19 |  |
| SOt055 | *tRNA-Lys-7* | tRNA-Lys-7 | -1.03 |  |
| SOt060 | *tRNA-Lys* | tRNA-Lys-8 | -1.01 |  |
| SOt063 | *tRNA-Ala-5* | tRNA-Ala-5 | -1.22 |  |
| SOt064 | *tRNA-Ile-3* | tRNA-Ile-3 | -1.06 |  |
| SOt065 | *tRNA-Glu-6* | tRNA-Glu-6 | -1.39 |  |
| SOt066 | *tRNA-Glu-5* | tRNA-Glu-5 | -1.24 |  |
| SOt067 | *tRNA-Glu-4* | tRNA-Glu-4 | -1.26 |  |
| SOt068 | *tRNA-Glu-3* | tRNA-Glu-3 | -1.28 |  |
| SOt069 | *tRNA-Glu-2* | tRNA-Glu-2 | -1.37 |  |
| SOt072 | *tRNA-Ala-3* | tRNA-Ala-3 | -1.31 |  |
| SOt073 | *tRNA-Val-5* | tRNA-Val-5 | -1.04 |  |
| SOt074 | *tRNA-Val-4* | tRNA-Val-4 | -1.06 |  |
| SOt075 | *tRNA-Val-3* | tRNA-Val-3 | -1.08 |  |
| SOt076 | *tRNA-Val-2* | tRNA-Val-2 | -1.21 |  |
| SOt077 | *tRNA-Val-1* | tRNA-Val-1 | -1.58 |  |
| SOt083 | *tRNA-Arg-8* | tRNA-Arg-8 | -1.09 |  |
| SOt084 | *tRNA-Ser-4* | tRNA-Ser-4 | -1.64 |  |
| SOt087 | *tRNA-Arg-5* | tRNA-Arg-5 | -1.11 |  |
| SOt088 | *tRNA-Arg-4* | tRNA-Arg-4 | -1.14 |  |
| SOt089 | *tRNA-Arg-3* | tRNA-Arg-3 | -1.03 |  |
| SOt090 | *tRNA-Ser-3* | tRNA-Ser-3 | -1.65 |  |
| SOt094 | *tRNA-Leu-3* | tRNA-Leu-3 |  | -1.1764 |
| SOt095 | *tRNA-Ala-2* | tRNA-Ala-2 | -1.22 |  |
| SOt096 | *tRNA-Ile-2* | tRNA-Ile-2 | -1.06 |  |
| SOt097 | *tRNA-Pro-3* | tRNA-Pro-3 | -1.05 |  |
| SOt098 | *tRNA-Pro-2* | tRNA-Pro-2 | -1.15 |  |
| SOt100 | *tRNA-Arg-2* | tRNA-Arg-2 | -1.01 |  |
| ABC transporters | | | | |
| SO0056 |  | Transport system substrate binding component |  | 1.05 |
| SO0070 | *natA* | ABC-type sodium efflux system ATPase component |  | 1.04 |
| SO0073 |  | ABC-type efflux system ATPase component |  | 1.11 |
| SO0074 |  | ABC transporter, permease protein | 1.06 | 1.14 |
| SO0525 | *rmrB* | Multidrug resistance protein | 1.05 | 1.36 |
| SO0821 | *macB* | Macrolide export ATP-binding/permease protein | 1.03 |  |
| SO0822 | *macC* | RND efflux system, outer membrane lipoprotein | 1.01 | 1.07 |
| SO0858 | *glyP* | Na(+)-linked D-alanine glycine transporter |  | 1.05 |
| SO1034 | *btuC* | Cobalamin uptake system permease component |  | 1.08 |
| SO1273 | *potI* | Putrescine transport system permease protein | 1.36 | 1.25 |
| SO1647 | *kefB* | Glutathione-gated K(+)-efflux system |  | 1.07 |
| SO1760 |  | AzlC family protein | 1.46 |  |
| SO1918 |  | Multidrug and toxin efflux protein MATE family |  | 1.06 |
| SO3485 | *emrD3* | Multidrug efflux pump |  | 1.03 |
| SO3674 | *hmuC* | Hemin ABC transporter, permease protein | 1.17 |  |
| SO3691 |  | Macrolide export system permease component 2 |  | 1.20 |
| SO3692 |  | Macrolide export system ATPase component |  | 1.02 |
| SO3694 |  | ABC transporter, permease protein | 1.03 | 1.50 |
| SO4447 | *modB* | Molybdenum transport system permease protein | 1.24 | 1.01 |
| SO4527 |  | Permease of the drug/metabolite | 1.11 | 1.21 |
| Other transporters | | | | |
| SO0057 | *ktrB* | Na-dependent K uptake membrane component |  | 1.25 |
| SO0058 | *ktrA* | Na-dependent K uptake NAD binding component |  | 1.15 |
| SO0157 |  | Proton:glutamate symporter DAACS family |  | 1.24 |
| SO0194 |  | Acyl transferase |  | 1.17 |
| SO0455 |  | alpha-ketoglutarate uptake system |  | 1.01 |
| SO0715 | *sorA* | SO3 dehydrogenase molybdopterin-binding subunit |  | 1.05 |
| SO0737 | *nicT* | TonB-dependent nickel receptor |  | 1.18 |
| SO0760 | *amt* | Ammonium transporter | 1.38 | 1.42 |
| SO1047 | *lrgA* | Holin-like protein CidA | 1.23 |  |
| SO1917 |  | Major facilitator superfamily transporter |  | 1.07 |
| SO2195 |  | Inter-alpha-trypsin inhibitor family protein |  | 1.31 |
| SO2523 |  | TonB-dependent receptor | 1.05 | 1.31 |
| SO2713 | *pnuT* | Predicted thiamin transporter | 1.15 | 1.13 |
| SO3503 | *nagP* | N-acetyl glucosamine transporter | 1.07 |  |
| SO4004 |  | Proton/sodium:glutamate symporter DAACS family |  | 1.03 |
| SO4050 |  | Putative transport system permease component |  | 1.12 |
| SO4081 | *puuP* | Putrescine uptake protein PuuP |  | 1.14 |
| SO4296 | *nupC* | Na-dependent nicotinamide ribose transporter |  | 1.20 |
| SO4339 |  | Transporter |  | 1.24 |
| Respiration | | | | |
| SO0259 | *ccmE* | Cytochrome c-type biogenesis protein |  | -1.26 |
| SO0260 | *ccmD* | Heme export system CcmE-interacting component |  | -1.32 |
| SO0261 | *ccmC* | ABC-type heme export system permease component 2 |  | -1.10 |
| SO0264 | *scyA* | Cytochrome c-type protein | -1.41 |  |
| SO0476 | *sirH* | Cytochrome c maturation periplasmic thioredoxin |  | 1.09 |
| SO0477 | *sirF* | cytochrome c maturation syste | 1.10 | 1.21 |
| SO0478 | *sirE* | Cytochrome c maturation system haem lyase subunit |  | 1.36 |
| SO0479 | *sirA* | Sulfite reductase SirA |  | 1.10 |
| SO0480 | *sirB* | Sulfurtransferase SirB |  | 1.20 |
| SO0481 | *sirI* | Peptidyl-prolyl cis-trans isomerase |  | 1.03 |
| SO0483 | *sirC* | 4Fe-4S ferredoxin SirC |  | 1.09 |
| SO0484 | *sirD* | Menaquinol oxidase |  | 1.15 |
| SO0485 | *nosL* | Copper uptake periplasmic chaperone component |  | 1.00 |
| SO0488 | *nosY* | C copper transport system permease component |  | 1.08 |
| SO0630 | *nosA* | TonB-dependent copper receptor |  | 1.01 |
| SO0714 |  | Periplasmic monoheme cytochrome c4 |  | 1.14 |
| SO0717 |  | Periplasmic monoheme cytochrome c4 |  | 1.55 |
| SO0845 | *napB* | Nitrate reductase cytochrome c550-type subunit | 1.28 |  |
| SO0846 | *napH* | Polyferredoxin NapH (periplasmic nitrate reductase) | 1.43 |  |
| SO0848 | *napA* | Periplasmic nitrate reductase precursor | 1.11 | 1.13 |
| SO0849 | *napD* | Periplasmic nitrate reductase chaperone |  | 1.12 |
| SO0904 | *nqrC* | Na(+)-translocating NADH-quinone reductase subunit C |  | -1.09 |
| SO0970 | *fccA* | periplasmic fumarate reductase | -1.43 | -1.97 |
| SO1233 | *torC* | TMAO reductase associated c-type cytochrome | 1.32 |  |
| SO1413 |  | Flavocytochrome c heme submit |  | 1.27 |
| SO1414 |  | Flavocytochrome c flavin subunit |  | 1.29 |
| SO1776 | *mtrB* | FeO respiratory outer membrane component |  | -1.36 |
| SO1777 | *mtrA* | FeO respiratory cytochrome c component |  | -1.51 |
| SO1778 | *mtrC* | FeO respiratory se cytochrome c component |  | -1.49 |
| SO1779 | *omcA* | D decaheme cytochrome c lipoprotein | -1.04 | -1.72 |
| SO1929 | *sdhB* | Succinate dehydrogenase iron-sulfur protein | -1.02 |  |
| SO2361 | *ccoP* | Cbb3-type cytochrome c oxidase subunit |  | -1.06 |
| SO2362 | *ccoQ* | Cytochrome c oxidase (cbb3-type) subunit CcoQ | -1.18 | -1.20 |
| SO2931 |  | Cytochrome c lipoprotein |  | 1.20 |
| SO3058 |  | Flavocytochrome c flavin subunit | 1.14 | 1.05 |
| SO3286 | *cydA* | Cytochrome d ubiquinol oxidase subunit I |  | -1.01 |
| SO3325 | *nrfJ* | Uncharacterized protein |  | -1.38 |
| SO3885 |  | AAA ATPase, central domain protein | 1.02 |  |
| SO4142 |  | Periplasmic monoheme cytochrome c |  | 1.29 |
| SO4144 | *otr* | Octaheme tetrathionate reductase |  | 1.17 |
| SO4202 | *tatA* | Twin-arginine translocation protein | -1.13 |  |
| SO4483 |  | Cytochrome b |  | 1.14 |
| SO4484 | *shp* | Monoheme cytochrome c |  | 1.13 |
| SO4568 | *nrfD* | Nitrite reductase quinol dehydrogenase component |  | 1.50 |
| SO4591 | *cymA* | Cytochrome c-type protein |  | -1.45 |
| SO4607 | *coxA* | Aa3 type cytochrome c oxidase subunit I |  | 1.03 |
| SO4608 | *ctaG* | Cytochrome oxidase biogenesis protein | 1.05 |  |
| SO4614 | *ctaB* | Protoheme IX farnesyltransferase |  | 1.10 |
| SO4694 | *torF* | TMAO reductase system outer membrane porin |  | 1.38 |
| SO4746 | *atpC* | ATP synthase epsilon chain | -1.16 |  |
| Carbohydrate metabolism | | | | |
| SO0425 | *aceF* | Acetyltransferase pyruvate dehydrogenase complex |  | -1.02 |
| SO1490 | *adhB* | Alcohol dehydrogenase | -1.13 | -1.06 |
| SO1493 | *malQ* | 4-alpha-glucanotransferase |  | 1.41 |
| SO1494 | *glgB* | 1,4-alpha-glucan branching enzyme |  | 1.15 |
| SO1495 | *glgX* | Glycogen debranching enzyme | 1.03 | 1.22 |
| SO1496 | *glgP* | Alpha-1,4 glucan phosphorylase |  | 1.31 |
| SO1498 | *glgC* | Glucose-1-phosphate adenylyltransferase |  | 1.39 |
| SO1499 | *glgA* | Glycogen synthase ADP-glucose transglucosylase |  | 1.06 |
| SO1518 | *lldG* | L-lactate dehydrogenase complex protein |  | -1.45 |
| SO1519 | *lldF* | L-lactate dehydrogenaseFe-Sur cluster-binding protein |  | -1.75 |
| SO1520 | *lldE* | L-lactate dehydrogenase complex protein |  | -2.17 |
| SO1677 | *ivdA* | 3-ketoacyl-CoA thiolase |  | -1.01 |
| SO1678 | *ivdB* | methylmalonate-semialdehyde dehydrogenase | -1.18 |  |
| SO1893 | *liuE* | Hydroxymethylglutaryl-CoA lyase | -1.09 |  |
| SO1931 | *sucB* | 2-oxoglutarate dehydrogenase complex | -1.00 | -1.01 |
| SO1932 | *sucC* | Succinyl-CoA ligase [ADP-forming] subunit beta |  | -1.02 |
| SO1933 | *sucD* | Succinyl-CoA ligase [ADP-forming] subunit alpha |  | -1.00 |
| SO2054 | *frmA* | S-(hydroxymethyl)glutathione dehydrogenase | 1.03 |  |
| SO2445 | *thiC* | Hydroxymethylpyrimidine phosphate synthase | 1.01 |  |
| SO2524 | *phyS* | beta-propeller phytase PhyS |  | 1.07 |
| SO2644 | *ppsA* | Phosphoenolpyruvate synthase |  | -1.18 |
| SO2912 | *pflB* | Pyruvate formate-lyase |  | -1.50 |
| SO3599 | *cysP* | Sulfate and thiosulfate binding protein | -1.15 |  |
| Biosynthesis of aminoacids | | | | |
| SO0276 | *argB* | Acetylglutamate kinase |  | 1.00 |
| SO0279 | *argH* | Argininosuccinate lyase |  | 1.10 |
| SO0818 | *metE* | Homocysteine methyltransferase | 1.13 | 1.32 |
| SO1361 | *aroF* | Phospho-2-dehydro-3-deoxyheptonate aldolase |  | 1.01 |
| SO1770 | *garK* | Glycerate kinase | 1.01 |  |
| SO2074 | *hisG* | ATP phosphoribosyltransferase | 1.11 | 1.20 |
| SO2767 | *asnB* | Asparagine synthetase [glutamine-hydrolyzing] | 1.02 |  |
| SO3986 | *lysC* | Aspartokinase |  | 1.11 |
| SO4245 | *argA* | N-acetylglutamate synthase | 1.01 | 1.11 |
| Metabolism of cofactors and vitamins | | | | |
| SO1031 | *cobC* | Alpha-ribazole-5-phosphate phosphatase |  | 1.02 |
| SO2444 | *thiDE* | Thiamine-phosphate synthase |  | 1.23 |
| SO2445 | *thiC* | Phosphomethylpyrimidine synthase |  | 1.34 |
| SO3924 | *hydX* | Fe hydrogenase assembly protein |  | 1.21 |
| SO3925 | *hydE* | Fe hydrogenase maturation rSAM protein |  | 1.06 |
| SO4626 | *bioH* | Pimeloyl-[acyl-carrier protein] methyl ester esterase |  | 1.10 |
| Chaperones | | | | |
| SO0052 | *secB* | Protein-export protein |  | -1.31 |
| SO0406 | *trxA* | Thioredoxin |  | -1.31 |
| SO0703 | *groES* | Heat shock protein 60 family co-chaperone | -1.37 | -1.16 |
| SO0704 | *groEL* | 60 kDa chaperonin |  | -1.49 |
| SO1126 | *dnaK* | Chaperone protein DnaK |  | -1.29 |
| SO1127 | *dnaJ* | Chaperone protein DnaJ |  | -1.01 |
| SO1197 | *ftsH* | ATP-dependent zinc metalloprotease |  | -1.00 |
| SO1793 | *tig* | Cell division trigger factor | -1.12 |  |
| SO2016 | *htpG* | Chaperone protein HtpG |  | -1.07 |
| SO2277 | *ibpA* | 16 kDa heat shock protein A |  | -1.18 |
| SO3577 | *clpB* | Chaperone protein ClpB |  | -1.10 |
| Curli | | | | |
| SO0866 | *csgB* | Minor curlin subunit |  | 1.55 |
| SO3685 | *csgG* | Curli secretion membrane lipoprotein component |  | 1.16 |
| SO3686 | *csgF* | Curli secretion apparatus component |  | 1.25 |
| SO4579 | *fimA* | P pilus subunit FimA |  | 1.08 |
| Flagella | | | | |
| SO3245 | *flgF* | Flagellar component |  | 1.14 |
| SO3250 | *flgB* | Flagellar basal body rod protein |  | 1.45 |
| SO3258 | *flgT* | Flagella assembly protein |  | 1.01 |
| DNA repair | | | | |
| SO0548 |  | Histone-like DNA-binding protein;SO0548 |  | -1.25 |
| SO1797 | *hupB* | DNA-binding protein HU-beta | -1.06 |  |
| SO2087 | *ihfA* | Integration host factor alpha subunit | -1.09 | -1.01 |
| SO2401 | *ihfB* | Integration host factor subunit beta |  | -1.55 |
| SO4267 | *hsdR* | Type I restriction-modification system subunit |  | 1.21 |
| SO4696 |  | DNA-3-methyladenine glycosylase | 1.04 |  |
| SOA0012 | *umuC* | Error-prone, lesion bypass DNA polymerase V | 1.11 | 1.01 |
| SOA0013 | *umuD* | Error-prone repair protein | 1.43 | 1.06 |
| Transcriptional regulators | | | | |
| SO0082 |  | Transcriptional activator of benzoate metabolism |  | 1.34 |
| SO0295 |  | Transcriptional regulator LysR family |  | 1.11 |
| SO0369 |  | Transcriptional regulator LysR family |  | 1.01 |
| SO0393 | *fis* | DNA-binding protein |  | -1.15 |
| SO0532 | *arsR* | Arsenite-sensing transcriptional repressor |  | 1.06 |
| SO0624 | *crp* | cAMP-responsive regulator of catabolite repression |  | -1.33 |
| SO0761 | *glnK* | regulatory protein for nitrogen assimilation GlnK |  | 1.23 |
| SO0817 | *metR* | Transcription regulator of Met biosynthesis MetR |  | 1.08 |
| SO0997 |  | Transcriptional regulator LysR family |  | 1.03 |
| SO1687 | *cueR* | Cu(I)-responsive transcriptional regulator | 1.19 |  |
| SO1898 | *liuR* | Transcriptional regulator LiuR of Leu degradation | -1.35 |  |
| SO2305 | *lrp* | Leucine-responsive regulatory protein | -1.10 |  |
| SO2519 |  | Transcriptional regulator AraC family |  | 1.34 |
| SO2787 | *cspA* | Cold shock protein CspA |  | -1.07 |
| SO3332 |  | Transcriptional regulator CopG family |  | 1.34 |
| SO3743 |  | Transcriptional regulator, AcrR family | -1.04 |  |
| SO3819 | *glnK* | Regulatory protein for nitrogen assimilation |  | 1.14 |
| Oxidative stress defense | | | | |
| SO0725 | *katG2* | Catalase-peroxidase 2 |  | 1.23 |
| SO1070 | *katB* | Catalase |  | 1.09 |
| SO4302 | *dnrN* | Repair of iron centers (RIC) protein |  | 1.54 |
| SO4405 | *katG1* | Catalase-peroxidase 1 |  | 1.01 |
| Two-components systems | | | | |
| SO3210 | *fliA* | RNA polymerase sigma factor for flagellar operon | -1.03 |  |
| SO0059 | *kdpE* | K+ channel response regulator |  | 1.08 |
| SO0060 | *kdpD* | Osmosensitive K+ channel histidine kinase | 1.07 | 1.38 |
| SO3209 | *cheY* | Chemotaxis signal transduction response regulator |  | -1.04 |
| SO4466 |  | Chemotaxis signal transduction |  | 1.19 |
| SO4638 |  | Histidine kinase |  | 1.11 |
| Type II secretion systems | | | | |
| SO4105 | *mshA* | MSHA pilin protein | -1.02 |  |
| SO0417 | *pilA* | Type IV pilin protein |  | -1.26 |
| SO4105 | *mshA* | MSHA major pilin subunit |  | -1.04 |
| Type I secretion systems | | | | |
| SO4319 | *aggB* | Type I protein secretion system MFP component |  | -1.14 |
| SO4320 | *aggA* | Type I protein secretion system secretin component |  | -1.37 |
| Porins |  |  |  |  |
| SO3896 | *omp35* | outer membrane porin, putative | -1.24 | -1.40 |
| SO1673 | *ompW* | Outer membrane protein |  | -1.37 |
| SO3545 | *ompAF* | Outer membrane porin |  | -1.30 |
| SO2194 | *pdsO* | Sortase system OmpA family protein |  | 1.16 |
| Transposase activity | | | | |
| SO2141 |  | iSSod16 transposase |  | 1.01 |
| SO3296 |  | ISSod12 transposase |  | 1.16 |
| SO3854 |  | ISSod12 transposase |  | 1.00 |
| SOA0016 |  | ISSod9, transposase | 1.51 | 1.01 |
| SOA0120 |  | ISSod12 transposase |  | 1.13 |
| SOA0167 |  | ISSod9, transposase | 2.14 | 1.01 |
| Cold shock | | | | |
| SO1648 |  | Cold shock protein | -1.15 |  |
| Heat shock | | | | |
| SO2728 | *htpX* | Protease HtpX homolog | -1.10 |  |
| Translation factors | | | | |
| SO3579 | *rluD* | Pseudouridine synthase |  | -1.03 |
| Stress proteins | | | | |
| SO3681 | *uspA* | Universal stress protein family |  | -1.35 |
| Fe acquisition | | | | |
| SO1829 |  | TPR protein, putative component of TonB system | -1.21 |  |
| Membrane stabilization | | | | |
| SO1295 |  | Outer membrane lipoprotein | -1.17 |  |
| Xenobiotics metabolism | | | | |
| SO4153 | *sye3* | NAD(P)H:flavin oxidoreductase |  | 1.29 |
| Nucleotides metabolism | | | | |
| SO2403 | *cmk* | Cytidylate kinase | -1.17 |  |
| Other transporters | | | | |
| SO3099 |  | Long-chain fatty acid transport protein | -1.01 |  |
| Ribosome biogenesis | | | | |
| SO1358 | *rimM* | 16S rRNA processing protein | -1.36 |  |
| Proteins degradation | | | | |
| SO0349 | *yfiB* | ATP-dependent serine peptidase S16 family |  | 1.18 |
| mRNA degradation | | | | |
| SO3783 | *rhlE* | ATP-dependent RNA helicase |  | 1.10 |
| Mu phage | | | | |
| SO0643 | *tnpA* | Mu phage transposase OrfA |  | 1.01 |
| SO0644 |  | Mu phage transposase OrfB |  | 1.14 |
| SO0661 |  | Mu phage transcriptional regulator |  | 1.41 |
| SO0666 | *gpE* | Mu phage large terminase subunit |  | 1.01 |
| SO0667 |  | Mu phage portal protein |  | 1.10 |
| SO0669 |  | phage virion morphogenesis protein | 1.05 |  |
| SO0675 |  | Mu phage major head subunit |  | 1.07 |
| SO0680 |  | Mu phage tape measure protein |  | 1.06 |
| SO1448 |  | Phage integrase family protein | 1.41 | 1.42 |
| SO1453 | *yfjZ* | Mu phage lytic protein |  | 1.60 |
| SO2654 |  | Mu phage transposase OrfA |  | 1.16 |
| SO2657 |  | Mu phage uncharacterized protein |  | 1.03 |
| SO2658 |  | Mu phage uncharacterized protein |  | 1.01 |
| SO2672 |  | Mu phage uncharacterized protein E18 |  | 1.05 |
| SO2673 |  | Mu phage uncharacterized protein |  | 1.35 |
| SO2674 | *com* | Mu phage Mom translational regulator |  | 1.04 |
| SO2678 |  | Mu phage uncharacterized protein |  | 1.13 |
| SO2684 | *gpl* | Mu phage protease |  | 1.54 |
| SO2687 |  | Mu phage uncharacterized protein |  | 1.50 |
| SO2701 | *pg46* | Mu phage uncharacterized protein |  | 1.16 |
| SO2703 | *gp48* | Mu phage phage morphogenesis protein |  | 1.06 |
| SOA0014 |  | Mobile element protein | 2.27 |  |
| Unclassified | | | | |
| SO0050 |  | Rhodanese-related sulfurtransferase | -1.09 |  |
| SO0071 |  | Hydrolase alpha/beta hydrolase fold family |  | 1.03 |
| SO0180 | *phyH* | Phytanoyl-CoA dioxygenase |  | 1.23 |
| SO0192 |  | Acteyltransferase GNAT family |  | 1.00 |
| SO0202 |  | Predicted membrane protein |  | 1.10 |
| SO0334 |  | Uncharacterized protein |  | -1.51 |
| SO0335 | *zapB* | Cell division protein ZapB |  | -1.10 |
| SO0440 |  | Cell surface immunomodulating metalloprotease |  | 1.04 |
| SO0455 |  | Alpha-ketoglutarate uptake permease component |  | 1.01 |
| SO0555 |  | Bifunctional diguanylate cyclase/phosphodiesterase |  | 1.05 |
| SO0585 | *sorA* | D-3-phosphoglycerate dehydrogenase | 1.02 | 1.05 |
| SO0659 |  | lysozyme, putative | 1.10 |  |
| SO0715 |  | Oxidoreductase, molybdopterin-binding | 1.05 |  |
| SO0727 |  | UPF0251 protein |  | 1.30 |
| SO0768 |  | NAD dependent epimerase/dehydratase protein |  | 1.04 |
| SO0867 |  | Collagenolytic serine protease |  | 1.13 |
| SO0877 |  | Acteyltransferase GNAT family |  | 1.26 |
| SO1053 |  | Phosphoenolpyruvate/pyruvate superfamily protein |  | 1.03 |
| SO1072 | *gbpA* | GlcNAc-binding protein A |  | 1.11 |
| SO1238 |  | Lysophospholipid acyltransferase |  | 1.12 |
| SO1239 |  | metallophosphoesterase | 1.14 |  |
| SO1249 |  | Peptidase U32 family |  | 1.24 |
| SO1395 |  | protein of unknown function DUF1294 | 1.05 |  |
| SO1447 |  | Retinol acyltransferase domain protein |  | 1.34 |
| SO1486 |  | 4-hydroxybenzoyl-CoA thioesterase family protein |  | 1.05 |
| SO1489 |  | Putative cytoplasmic protein |  | -1.36 |
| SO1492 |  | peptidylprolyl cis-trans isomerase lipoprotein |  | 1.18 |
| SO1587 |  | Predicted membrane protein |  | 1.16 |
| SO1694 |  | FAD-binding protein |  | 1.09 |
| SO1958 |  | Glyoxylase-like domain protein |  | 1.13 |
| SO1975 |  | Zinc carboxypeptidase-related protein |  | 1.14 |
| SO1991 |  | Uncharacterized protein |  | -1.17 |
| SO2101 |  | Predicted lipoprotein |  | 1.08 |
| SO2155 |  | Glyoxalase family protein |  | 1.33 |
| SO2195 |  | Inter-alpha-trypsin inhibitor domain protein | 1.10 |  |
| SO2747 | *pal* | Peptidoglycan-associated lipoprotein |  | -1.08 |
| SO2821 | *yecH* | Probable metal-binding protein |  | -1.28 |
| SO2836 |  | Patatin-like phospholipase |  | 1.00 |
| SO2851 |  | D-glycero-D-manno-heptose 1,7-bisphosphate phosphatase | -1.00 |  |
| SO2930 |  | Bifunctional pectinolytic enzyme/cytochrome c |  | 1.10 |
| SO3065 |  | Colicin V production protein | -1.06 |  |
| SO3085 |  | Predicted periplasmic protein |  | 1.39 |
| SO3106 | *sapSH* | Extracellular serine alkaline protease |  | 1.04 |
| SO3284 | *ybgT* | Protein YbgT |  | -1.23 |
| SO3325 | *nrfJ* | NrfJ-related protein | -1.40 |  |
| SO3561 |  | Membrane protein |  | 1.45 |
| SO3765 |  | PspA/IM30 family protein |  | -1.02 |
| SO3830 |  | Protein SirB2 | -1.02 |  |
| SO3870 | *dsbI* | Putative protein-disulfide oxidoreductase |  | 1.00 |
| SO3885 |  | AAA ATPase family protein |  | 1.22 |
| SO3996 |  | Predicted inner membrane protein |  | 1.29 |
| SO4050 |  | Permease of the major facilitator superfamily | 1.34 |  |
| SO4079 |  | UPF0307 protein |  | -1.09 |
| SO4149 |  | RTX toxin, putative | 1.04 |  |
| SO4152 |  | SnoaL family protein |  | 1.53 |
| SO4169 |  | Deoxyribodipyrimidine photolyase-related protein |  | 1.05 |
| SO4194 |  | Glycosyl transferase |  | 1.59 |
| SO4226 | *ftsL* | Cell division protein |  | -1.03 |
| SO4280 |  | CBS domain containing protein |  | -1.08 |
| SO4295 |  | Quinone oxidoreductase family protein |  | 1.28 |
| SO4311 | *cyaY* | Protein CyaY |  | -1.08 |
| SO4317 | *bpfA* | Biofilm-promoting protein BpfA |  | -1.33 |
| SO4321 |  | Pal-like T1SS-linked outer membrane lipoprotein |  | -1.35 |
| SO4334 | *creD* | Membrane protein |  | 1.04 |
| SO4335 | *pgpB* | Phosphatidylglycerophosphatase B |  | 1.44 |
| SO4473 |  | Outer membrane protein A precursor | -1.13 |  |
| SO4508 | *fdhX* | Formate dehydrogenase accesory protein |  | -1.14 |
| SO4520 |  | Coproporphyrinogen III oxidase oxygen-independent |  | -1.00 |
| SO4539 |  | Extracellular peptidase family S8 |  | 1.01 |
| SO4543 |  | Putative alpha-dextrin endo-1 6-alpha-glucosidase |  | 1.12 |
| SO4590 |  | Isochorismatase family protein |  | 1.10 |
| SO4625 |  | Predicted phosphoribosyltransferase ComF family |  | 1.40 |
| SO4636 |  | Predicted lipoprotein |  | 1.35 |
| SO4646 |  | Propeptide, PepSY amd peptidase M4 precursor | 1.07 |  |
| SO4690 |  | Glycosyl transferase family 83 |  | 1.16 |
| SO4701 |  | Predicted periplasmic protein |  | 1.20 |
| SO4764 |  | Predicted lipoprotein |  | 1.05 |
| SO4810 | *leuL* | LeuABCD operon attenuation leader peptide |  | -1.17 |
| SOA0023 | *higA* | Antitoxin | -1.26 |  |
| SOA0060 |  | Acetyltransferase | -1.04 |  |
| SOA0095 | *parA* | Plasmid partition protein |  | -1.14 |
| SOA0096 | *parB* | Plasmid partition protein |  | -1.15 |
| SOA0122 |  | Protein co-occuring with Mo cofactor biosynthesis protein B | -1.11 |  |
| SOA0122 | *ygiW* | Plasmid-encoded periplasmic BOF domain protein |  | -1.02 |
| SOm003 |  | tmRNA | -1.50 |  |
| Uncharacterized proteins | | | | |
| SO0111 |  | Uncharacterized protein |  | -1.13 |
| SO0115 |  | Uncharacterized protein |  | -2.22 |
| SO0116 |  | Uncharacterized protein |  | -1.43 |
| SO0318 |  | Uncharacterized protein |  | -1.37 |
| SO0371 |  | Uncharacterized protein |  | -1.08 |
| SO0384 |  | Uncharacterized protein |  | -2.66 |
| SO0439 |  | Uncharacterized protein |  | -1.10 |
| SO0458 |  | Uncharacterized protein |  | -1.31 |
| SO0503 | *arsP* | Uncharacterized protein |  | -1.04 |
| SO0543 |  | Uncharacterized protein |  | -1.92 |
| SO0595 |  | Uncharacterized protein |  | -2.11 |
| SO0745 |  | Uncharacterized protein |  | -1.93 |
| SO0753 |  | Uncharacterized protein |  | -1.20 |
| SO0844 |  | Uncharacterized protein |  | -1.07 |
| SO1157 |  | Uncharacterized protein |  | 1.32 |
| SO1379 |  | Uncharacterized protein |  | 1.10 |
| SO1450 |  | Uncharacterized protein |  | 1.20 |
| SO1454 |  | Uncharacterized protein |  | 1.03 |
| SO1953 |  | Uncharacterized protein |  | 1.01 |
| SO2143 |  | Uncharacterized protein |  | 1.12 |
| SO2682 |  | Uncharacterized protein |  | 1.04 |
| SO3086 |  | Uncharacterized protein |  | 1.01 |
| SO3504 | *nagX* | Uncharacterized protein |  | 1.11 |
| SO3621 |  | Uncharacterized protein |  | 1.22 |
| SO3682 |  | Uncharacterized protein |  | 1.50 |
| SO3704 |  | Uncharacterized protein |  | 1.05 |
| SO3764 |  | Uncharacterized protein |  | 1.06 |
| SO3886 |  | Uncharacterized protein |  | 1.02 |
| SO3887 |  | Uncharacterized protein |  | 1.09 |
| SO3891 |  | Uncharacterized protein |  | 1.00 |
| SO4126 |  | Uncharacterized protein |  | 1.09 |
| SO4134 |  | Uncharacterized protein |  | 1.59 |
| SO4143 |  | Uncharacterized protein |  | 1.01 |
| SO4430 |  | Uncharacterized protein |  | 1.33 |
| SO4530 |  | Uncharacterized protein |  | 1.58 |
| SO4558 |  | Uncharacterized protein |  | 1.04 |
| SO4844 |  | Uncharacterized protein |  | 1.11 |
| SOA0007 |  | Uncharacterized protein |  | 1.18 |
| Hypothetical proteins | | | | |
| SO0554 |  | hypothetical protein | -1.11 |  |
| SO0660 |  | Hypothetical protein | 1.03 |  |
| SO0680 |  | Hypothetical protein | 1.14 |  |
| SO0691 |  | hypothetical protein | -1.08 |  |
| SO0788 |  | hypothetical protein | -1.08 |  |
| SO1447 |  | Hypothetical protein | 1.01 |  |
| SO1450 |  | Hypothetical protein | 1.10 |  |
| SO1453 |  | Hypothetical protein | 1.19 |  |
| SO1835 |  | hypothetical protein | -1.15 |  |
| SO1958 |  | Hypothetical protein | 1.11 |  |
| SO1984 |  | hypothetical protein | -1.36 |  |
| SO2010 |  | hypothetical protein | -1.34 |  |
| SO2273 |  | hypothetical protein | -1.19 |  |
| SO2391 |  | hypothetical protein | -1.16 |  |
| SO2687 |  | Hypothetical protein | 1.08 |  |
| SO2773 |  | Hypothetical protein | 1.50 |  |
| SO3047 |  | Hypothetical protein | 1.00 |  |
| SO3733 |  | hypothetical protein | -1.07 |  |
| SO3886 |  | Hypothetical protein | 1.42 |  |
| SO3996 |  | Hypothetical protein | 1.02 |  |
| SO4044 |  | Hypothetical protein | 1.11 |  |
| SO4126 |  | Hypothetical protein | 1.41 |  |
| SO4429 |  | Hypothetical protein | 1.82 |  |
| SO4430 |  | Hypothetical protein | 1.53 |  |
| SO4651 |  | hypothetical protein | -1.52 |  |
| SO4788 |  | Hypothetical protein | 1.01 |  |
| SO4798 |  | hypothetical protein | -1.65 |  |
| SOA0007 |  | Hypothetical protein | 1.01 |  |
| SOA0008 |  | Hypothetical protein | 1.42 | 1.18 |
| SOA0009 |  | Hypothetical protein | 1.66 |  |
| SOA0014 |  | Hypothetical protein |  | 1.38 |
| SOA0015 |  | Hypothetical protein | 1.47 | 2.09 |
| SOA0042 |  | hypothetical protein | -1.04 |  |
| SOA0047 |  | hypothetical protein | -1.19 |  |
| SOA0127 |  | Hypothetical protein | 1.47 |  |
| SOA0132 |  | hypothetical protein | -1.25 |  |
| SOA0157 |  | hypothetical protein | -1.20 |  |
